# Naphthalimide‐Buckybowl Tweezer for Selective Recognition of Fullerene C_70_

**DOI:** 10.1002/chem.202500773

**Published:** 2025-03-26

**Authors:** Swapnil Ghule, Konstantin O. Korenkov, Dmitry I. Sharapa, Konstantin Y. Amsharov, Evgeny A. Kataev, Alexander S. Oshchepkov

**Affiliations:** ^1^ Department of Chemistry and Pharmacy Friedrich‐Alexander‐University Erlangen‐Nürnberg Nikolaus‐Fiebiger‐Straße 10 91058 Erlangen Germany; ^2^ Institute of Chemistry artin‐Luther‐University Halle‐Wittenberg 06120 Halle Germany; ^3^ Institute of Catalysis Research and Technology Karlsruhe Institute of Technology Hermann‐von‐Helmholtz‐Platz 1 76344 Eggenstein‐Leopoldshafen Germany

**Keywords:** buckybowl, dispersion interactions, fullerene, molecular tweezers, naphthalimide

## Abstract

Supramolecular tweezers‐like receptors represent a simple and efficient approach for the molecular recognition of fullerenes. Straightforward synthesis and easy fine‐tuning of their geometry are the advantages that allow one to achieve strong binding and specific selectivity. However, the use of buckybowls in constructing tweezers and incorporating fluorescent dyes is still underexplored. To achieve this goal, we have designed mono‐ and di‐substituted receptors by attaching indacenopicene to a naphthalimide dye. The tweezers‐like receptor shows the highest selectivity for C_70_ with an affinity of 2150 M^−1^, which is about 50‐fold stronger than the recognition of C_60_. DFT and NMR data indicate that the preferred binding mode involves the ellipsoidal C_70_ molecule coordinating with buckybowls at its poles. In this arrangement, the naphthalimide core establishes two CH–π interactions with the fullerene. The results indicate that conjugating buckybowls with naphthalimides in a suitable design presents a promising method for selective binding and fine‐tuning photoinduced electron transfer in the host–guest complex.

## Graphical abstract

Tweezer receptors incorporating bowl‐shaped binding sites have the potential to achieve better complementarity for fullerenes than the planar ones. Towards this goal, two molecules of indacenopicene were connected to the naphthalimide dye. Experimental studies revealed strong selectivity of the receptor for C_70_. This selectivity was rationalized by structural complementarity and the involvement of CH–π interactions. Institute and/or researcher Twitter usernames: @EAKataev, @UniFAU, @Alexand04121116

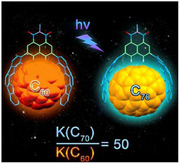

## 1 Introduction

Molecular tweezers are non‐cyclic supramolecular host molecules that provide effective noncovalent host‐guest interactions through π–π interactions and hydrogen bonds.^[^ ^1^, ^2^, ^3^ ^]^ Along with their structural flexibility, this has enabled a myriad of applications, ranging from selective guest recognition,^[^ ^4^, ^5^ ^]^ molecular switchers and machines,^[^ ^6^, ^7^ ^]^ to the use of these compounds in numerous preclinical disease models.^[^ ^3^, ^8^ ^]^ Nevertheless, selective binding of curved and spherical organic molecules is still challenging.^[^ ^9^ ^]^ As a spherical model, fullerene C_60_ is the most studied and has many intrinsic properties.^[^ ^10^ ^]^ By leveraging the electron‐acceptor properties of fullerenes and the electron‐donor characteristics of molecular tweezers, supramolecular donor‐acceptor‐donor (D‐A‐D) systems can be obtained upon binding with C_60_ and C_70_.^[^ ^11^, ^12^, ^13^ ^]^ Such systems can create nonlinear optical (NLO) materials whose properties are primarily characterized by optical coefficients and response speed.^[^ ^14^ ^]^ Unlike traditional organic single‐component materials, organic charge transfer (CT) complexes composed of donor and acceptor molecules achieve ultrafast responses and resonance enhancement through rapid intermolecular CT, improving optical coefficients.^[^ ^15^ ^]^ For this purpose, researchers have most intensively studied fullerene complexes with porphyrin tweezers.^[^ ^5^, ^16^ ^]^ However, the lack of geometric complementarity between planar porphyrin and spherical fullerene significantly decreases the binding constants and spatial electronic communication.^[^ ^11^ ^]^

To overcome this problem the use of bowl‐shaped molecules has been proposed.^[^ ^17^ ^]^ The host–guest chemistry of buckybowls and fullerenes is driven by the concave–convex π–π interactions arising from the interaction between the inner face of the buckybowl and the outer face of the pseudo‐spherical fullerene due to the asymmetrical nature of their π orbitals.^[^ ^18^, ^19^, ^20^, ^21^ ^]^ The association constants of fullerenes with pristine corannulene are small, and the selectivity is primarily determined by the host‐guest complementarity.^[^ ^17^ ^]^ Despite some success in creating corannulene tweezers,^[^ ^22^, ^23^, ^24^, ^25^, ^26^, ^27^ ^]^ systems based on other bowl‐shaped molecules remain extremely rare.^[^ ^11^, ^28^ ^]^ This is directly related to the high reaction barriers that must be overcome to synthesize non‐planar structures.^[^ ^29^, ^30^ ^]^ Tweezers‐like hosts for fullerene recognition have attracted much attention recently.^[^ ^2^, ^5^ ^]^ These systems are exciting in creating cascade photoinduced charge or electron transfer assemblies. Some of the reported tweezers for fullerenes show moderate^[^ ^11^, ^19^, ^31^, ^32^, ^33^ ^]^ or good selectivity for C_70_ with *K*(C_70_)/*K*(C_60_) around 10,^[^ ^11^, ^28^, ^32^, ^34^, ^35^, ^36^, ^37^, ^38^, ^39^ ^]^ or even 200.^[^ ^13^ ^]^ The binding data for the tweezers‐like receptors was obtained in most cases by UV‐Vis or fluorescence titrations. Our previous work reported a buckybowl catcher bearing the 1,3‐phenylene spacer, which shows moderate binding affinity and low C_70_/C_60_ selectivity.^[^ ^28^ ^]^ In the present study, we constructed a buckybowl‐tweezer, in which naphthalimide bears two buckybowls. This dye serves as an acceptor fluorescent subunit and a larger spacer that favors selectivity for C_70_. The binding studies revealed that the buckybowl building block binds fullerenes with 10^2^ M^−1^ affinity in a highly competitive tetrachloroethane solution, while the catcher has a strongly enhanced affinity for C_70_ (10^3^ M^−1^). To our knowledge, the designed receptor has one of the highest selectivity for C_70_ among those reported in the literature. As revealed from ^1^H NMR titrations, *K*(C_70_)/*K*(C_60_) binding selectivity is 50. DFT calculations support the geometrical complementarity of the receptor and C_70_ structure that coordinates with its poles to buckybowls. The donor–acceptor systems that bind fullerenes represent exciting targets for elucidating light‐induced charge‐transfer processes.

## 2 Results and Discussion

### 2.1 Design and synthesis

**Scheme 1:**
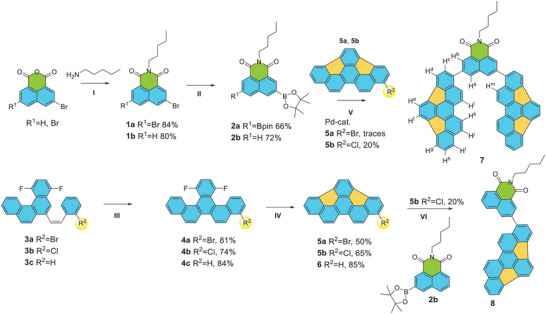
Synthetic routes toward tweezer **7** and reference compound **8**. Reaction conditions: (I) Ethanol, 80 °C, 12 h reflux; (II) Pd(dppf)Cl_2_, 1,4 Dioxane, B_2_Pin_2_ = bis(pinacolato)diboron, 100°C, 24 h; (III) hν, I_2_, propylene oxide, cyclohexane; (IV) γ‐Al_2_O_3_, vacuum, 240°C; (V, VI) Pd_2_(dba)_3_, XPhos, K_2_CO_3_, toluene, microwave irradiation, 110°C, 16 h.

Molecular tweezers are based on two main components: a binding site and a spacer.^[^ ^40^ ^]^ The interconnection of these components, their geometric matching, and the rigidity of the structure play an exceptional role in the effective recognition and binding of a guest.^[^ ^41^ ^]^ In the present study, a fluorescent naphthalimide dye was chosen as the spacer. Naphthalimides were used in many applications by our group and others, thanks to their good fluorescent properties and synthetically accessible derivatives.^[^ ^42^, ^43^, ^44^ ^]^ Naphthalimide might play several roles in the tweezer: (i) prevent self‐association of the recognition sites, (ii) orient buckybowls rigidly to recognize spherical guests, (iii) provide the necessary spacing between components,^[^ ^19^ ^]^ (iv) provide a fluorescence response upon binding to the fullerene, and (v) could potentially participate in electron transfer processes upon photoexcitation. A buckybowl – indacenopicene – extended curved aromatics with a large contact surface serves as a binding site for fullerenes.^[^ ^45^, ^46^ ^]^ We synthesized the designed tweezer (catcher) according to the synthetic route shown in Scheme 1.

Compound **8**, containing only one buckybowl and compound **6**, served as reference compounds in our study. We began our synthetic scheme with the preparation of mono‐ and dibrominated naphthalic anhydrides **1b** and **1a, respectively**.^[^ ^47^, ^48^ ^]^ The naphthalimide derivatives **2a** and **2b** were obtained via the acylation reaction of hexylamine with bromonaphthalic anhydride derivatives, followed by the Miyaura borylation reaction.^[^ ^47^ ^]^

For the synthesis of buckybowl precursors **5a,b**, we employed the same procedure previously described by us, but with variations in substituents.^[^ ^49^ ^]^ During the Mallory photocyclization, partial loss of bromine atoms was observed; however, the target compounds **4a–c** were obtained in good yields. In the next step, we carried out a cyclodehydrofluorination reaction, successfully yielding the buckybowls **5a,b** and **6**.

The Suzuki cross‐coupling reaction of 4‐bromo‐13,16‐difluorobenzo[s]picene (**5a**) with naphthalimide derivative **2a** resulted in a product formation only in trace amounts. Such a low yield is likely because of the harsh reaction conditions, leading to bromine elimination and the formation **6**. However, modifying the reaction conditions did not improve the yield, even under milder conditions.

To address this issue, we synthesized a chloro‐derivative of indacenopicene (**5b**), which successfully reacted with naphthalimide **2a** to afford compound **7** in 20% yield. The formation of the monosubstituted product was observed only in trace amounts. Therefore, the monosubstituted naphthalimide derivative **2b** was used in a cross‐coupling reaction with **5b**, resulting in the formation of compound **8** in 20% yield.

Notably, the synthetic route using chloro‐derivatives of buckybowls proved to be preferable, as the yield of the chloro‐derivative in the Mallory photocyclization reaction was also higher. This observation further supports the reduced probability of dehalogenation under UV‐light irradiation.^[^ ^50^, ^51^ ^]^

Along with tweezer **7**, the mono‐substituted analogue **8** served as a reference compound in terms of comparison of their binding properties toward fullerenes. The binding properties of **8** were expected to disclose how two buckbowls contribute to the binding selectivity.

### 2.2 Photochemical and binding properties

**Figure 1:**
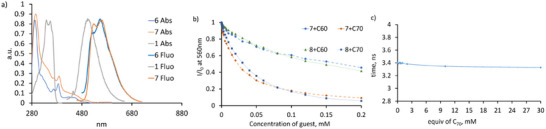
(a) Normalized absorption and fluorescence spectra of starting naphthalimide and indacenopicene, and **7**. (b) Fluorescence quenching observed by the addition of fullerenes to **7** and **8** (1 µM, in tetrachloroethane). (c) Lifetime measurements of **7** in the presence of different amounts of C_70_, as determined from time‐correlated single photon counting (TCSPC).

According to UV‐Vis measurement, compound **7** has two absorption bands corresponding to the overlapping absorption of **6** and naphthalimide **1**. Receptor **7** can be excited in this region, producing the emission in the region 490–670 nm with maxima at 527 and 560 nm (Figure 1a) and a determined quantum yield of 6.4%. The emission spectrum of indacenopicene **6** is in the same region, showing a 554 nm band.^[^ ^52^ ^]^ As can be seen in Figure 1a, the emission of **7** combines the emission of naphthalimide and indacenopicene. Due to the close emission, it is, however, difficult to conclude if the energy is transferred to one of the motifs.

**Figure 2:**
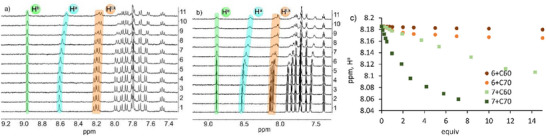
(a and b) ^1^H NMR titration of **7** with C_60_ and C_70_, respectively. (c) H^a^ (for **7**) and H^l^ (for **6**) proton shifts induced by the addition of fullerenes to the tweezer and buckybowl, respectively.

**Figure 3:**
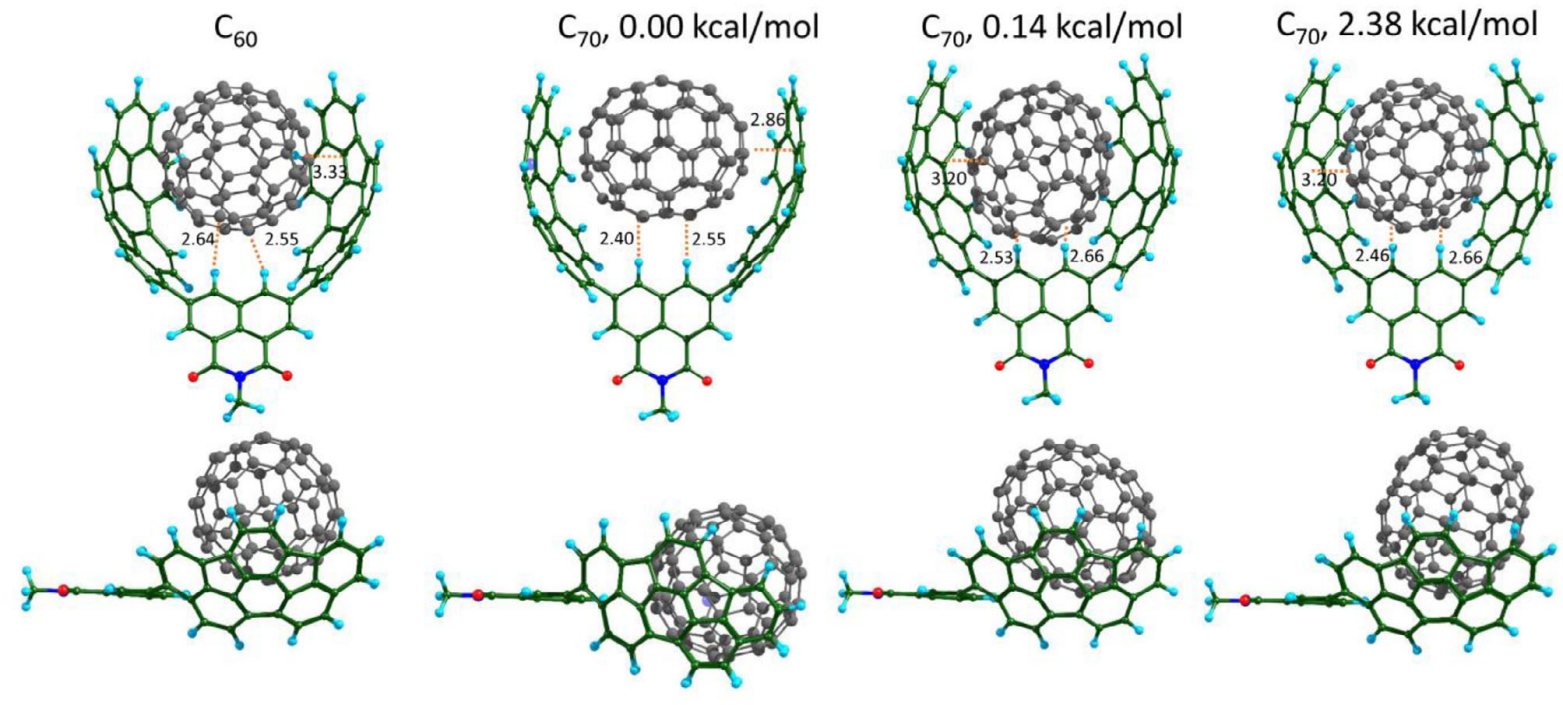
DFT optimized molecular structures of the supramolecular complexes of tweezer **7** with C_60_ and C_70_ (front and side views). The relative energies of the complexes and shortest distances are also shown.

The previously reported buckybowl catcher demonstrated low UV‐Vis and fluorescence changes upon adding fullerenes, likely due to the absence of a good fluorophore and relatively weak binding properties in a highly competitive solvent like o‐DCB.^[^ ^28^ ^]^ It is well known that combining low affinity with the inner filter effect humpers the accurate estimation of binding constants.^[^ ^53^ ^]^ However, our new naphthalimide catcher showed much stronger quenching in the presence of fullerenes C_60_ and C_70_ with apparent binding constants in tetrachloroethane 5000 M^−1^ and 40000 M^−1^, respectively (Figure 1a,b). Time‐resolved measurements of **7** in the presence of C_70_ showed no changes in the lifetime of the catcher (3.4 ns, Figure 1c), indicating the static quenching, that is, the formation of the supramolecular complex in the excited state. To determine the exact binding constants, we conducted ^1^H NMR titration experiments in C_2_D_2_Cl_4_. Special attention should be given to the proton shifts in the 4^th^ and 5^th^ (H^a^) positions of the naphthalimide. In the DFT optimized structure of complex **7**•C_70_, these protons are directed into the bound fullerenes forming H–π – interactions and, hence, should undergo considerable shifts upon fullerene binding (Figure 3).^[^ ^54^ ^]^ Analysis of the NMR data obtained in tetrachloroethane confirmed our suggestion. As shown in Figure 2a, adding C_60_ induces a substantial shift of H^a^ and protons H^m,k,l^ and H^d^ located in the bowl fragment close to the naphthalimide. In the case of C_70_ titration (Figure 2b), the shifts are stronger, and the signals start to broaden in the presence of an excess of the fullerene. We observed the precipitation of the complex after adding more than 20 equiv of C_70_. Since the proton signals were still visible, it was possible to calculate the binding constants. The fitting analysis shows that **7** binds C_60_ and C_70_ with binding constants 43 and 2147 M^−1^ (Table 1). These values correspond to the selectivity of about 50 in favor of C_70_ recognition.

**Table 1:** Binding constants (M^−1^) of 5–8 with fullerenes as determined from ^1^H NMR titrations in C_2_D_2_Cl_4_.

Guest	6	7	8
C_60_	140 ± 4	43 ± 2	314 ± 2
C_70_	668 ± 6	2147 ± 2	555 ± 2

To understand the origin of selectivity, we performed ^1^H NMR titration with buckybowl **6** and its conjugate **8**. We found that both compounds can also bind fullerenes in a tetrachloroethane solution. However, the selectivity between C_60_ and C_70_ is considerably lower than that of **7**. As can be seen in Table 1, the C_70_ selectivity between **6** and **8** are around 5‐ and 2‐fold, respectively. The comparison of the binding constants indicates that the incorporation of two buckybowls into the tweezer dramatically increases the selectivity for C_70_. Likely, the distance between the two buckybowls matches perfectly the size of C_70,_ and this parameter determines the selectivity. It is also possible that the free rotation of buckybowl through the C–C bond that connects it to the naphthalimide core hampers efficient coordination of C_60_. It can be suggested that the introduction of a substituent in the 1^st^ position of indacenopicene produces steric hindrance for the recognition of fullerenes. We performed temperature‐dependent ^1^H NMR spectra, which showed that the rotation of buckybows is present at room temperature and can be frozen only below ‐40˚C (Figure S39). At ‐40˚C, the signals start to broaden and coalesce. In order to obtain more structural information on how the binding of C_60_ and C_70_ differs from each other, we conducted DFT calculations (r^2^SCAN‐3c composite method that includes triple‐zeta quality basis set, D4 dispersion correction, and gCP correction^[^ ^55^ ^]^ by using ORCA 5.0.4 software^[^ ^56^ ^]^). For simplicity, we truncated the hexyl chain to a methyl group within the modeling. Figure 3 shows the most energetically favorable conformations for the complexes with fullerenes. The side views (bottom snapshots) show that in the case of C_60_ binding, the participation of CH–π interactions between the naphthalimide ring and the fullerene is less pronounced. In the case of C_70_ complex, CH‐protons of the naphthalimide are perpendicular to the long axis of C_70_ and point exactly into the center of the fullerene. The calculations clearly show that the distance between two buckybowls matches better the C_70_ recognition via coordination of the fullerene at both poles. The exact binding mode of C_70_ fullerene is often a question of interest. Sometimes it can be answered by X‐ray,^[^ ^57^ ^]^ in another recent case a clear answer can be obtained from DFT‐modelling.^[^ ^58^ ^]^ Our structure optimizations yielded, in sum, three different binding modes. The most favorable binding mode contains C_70,_ orienting with the poles towards buckybowls or perpendicular to them (*cf*. Supporting Information and Figure 3). Two binding modes are nearly equal in energy, while the third structure is less favorable, being 2 kcal/mol higher. Therefore, the calculations indicate that coordination with poles is more advantageous and can explain the observed selectivity.

In the case of C_60_ binding, the buckybowls must further rotate to accommodate a guest of a smaller size. According to the DFT calculations, the binding of C_70_ is 3.65 kcal/mol more favorable than that of C_60_. Thus, the supramolecular complex with C_70_ combines CH‒π, π‒π interactions, and a certain degree of shape selectivity that helps to favor the particular binding mode. Side‐views in Figure 3 reveal that CH‐protons of the naphthalimide are directed to the fullerene only in the most favorable structure with the CH‐fullerene shortest distances 2.40 and 2.55 Å.

## 3 Conclusion

In conclusion, we have synthesized a new receptor for fullerenes by connecting a fluorescent naphthalimide dye with a buckybowl indacenopicene via a palladium‐catalyzed cross‐coupling reaction. The spectroscopic investigations showed that the compound emits in the same region as the buckybowl building block. The addition of fullerenes strongly quenches the fluorescence of the receptor. According to the NMR binding studies, the receptor binds C_70_ with an affinity 50‐fold stronger than that for C_60_ in a tetrachloroethane solution. This affinity originates from the matching distance between the buckybowls and two poles of ellipsoidal C_70_. Unlike the interaction with C_60_, the complexation of C_70_ involves two additional CH‒π interactions between the protons in the 4^th^ and 5^th^ position of the naphthalimide core and the fullerene. This type of binding offers further exploration of site‐selective non‐covalent interactions with fullerenes and other polycyclic aromatic compounds. Combining fluorescent dye with buckybowls, which subsequently recognize fullerenes, represents a promising strategy to realize electron‐ or charge‐transfer events in supramolecular complexes with fullerenes under light excitation. This work is currently in progress.

## Supporting Information

The authors have cited additional references within the Supporting Information.^[^ ^28^, ^47^, ^49^, ^59^ ^]^

## Conflict of Interest

The authors declare no conflicts of interest.

## Supporting information



Supporting Information

## Data Availability

The data that support the findings of this study are available in the supplementary material of this article.
