# Supplementary material for: Naphthalimide‐Buckybowl Tweezer for Selective Recognition of Fullerene C70
Source: Chemistry. 2025 Mar 26;31(24):e202500773. doi: 10.1002/chem.202500773 (PMC12043031; doi:10.1002/chem.202500773)
Supplement: Supplementary file 1 — Supporting Information [file CHEM-31-e202500773-s001.pdf]

Supporting Information

Table of Contents

1. Materials and Methods 1
2. Synthetic Procedure 2
3. NMR Spectra 9
4. UV/vis 20
5. Fluorescence Titrations 21
6. NMR Titrations 23
7. DFT Calculations 27
8. Reference 43

1. Materials and Methods

All chemicals and solvents were purchased in reagent grade from commercial suppliers (Acros®, Sigma-Aldrich® or Fluka®, Fluorochem®, Merck®, ChemPur®) and used as received, unless otherwise specified. PURALOX SBa-200® was used for cyclodehydrofluorination reaction procedure. Solvents in HPLC grade were purchased from VWR® and Sigma-Aldrich®.

Flash column chromatography was performed on an Interchim PuriFlash XS420 using flash grade silica gel from (Machery-Nagel 60 M (40–63 mm, deactivated)).

NMR spectra were recorded on a Bruker Avance 400 at 400 MHz (^{1}H NMR) and 100 MHz (^{13}C NMR) and 600 at 600 MHz (^{1}H NMR) and 151 MHz (^{13}C NMR) respectively. The signals were referenced to residual solvent peaks (in parts per million (ppm) ^{1}H: $CDCl_3$, 7.27 ppm; CD_2Cl_2, 5.32 ppm; $C_2D_2Cl_4$, 6.00ppm. ^{13}C: $CDCl_3$, 77.0 ppm; CD_2Cl_2, 53.84 ppm; $C_2D_2Cl_4$, 73.78ppm). Coupling constants were assigned as observed. The obtained spectra were evaluated with the program MestReNova.

(MA)LDI-MS spectra were recorded on a Shimadzu Biotech AXIMA Confidence MALDI-TOF.

DCTB matrix (trans-2-[3-(4-tert-Butylphenyl)-2-methyl-2-propenylidene]malononitrile)

High resolution APPI spectra were recorded on a Bruker ESI TOF maXis 4G instrument. The data was evaluated with the program Bruker Compass Data Analysis 4.2.

HPLC measurements were performed on a Shimadzu Prominence Liquid Chromatograph LC-20AT with communication bus module CBM-20A, diode array detector SPDM20A, the degassing unit DGU-20A5 R, column oven CTO-20AC or CTO-20A, respectively and with auto sampler SIL- 20A HT. For separation a Cosmosil 5-PBR column (4.6 mm x 250 mm) from Nacalai Tesque was used. As eluent a toluene/MeOH/ or toluene/1,2-dichlorobenzene mixture or pure toluene or 1,2-dichlorobenzene was used (UV/Vis detection). The data was evaluated with the programs Shimadzu LC solution and Shimadzu LabSolutions.

UV/vis spectroscopy was carried out on a Varian Cary 5000 UV/Vis-NIR spectrometer. The spectra were measured in the indicated solvents in quartz cuvettes (edge length = 1 cm) at room temperature. The absorption maxima λmax are given in nanometer [nm].

Fluorescence spectroscopy was recorded on a FluoroMax 4 (Horiba) with temperature control. The samples were measured in 1 cm quartz cuvettes (Hellma) at room temperature. Emission maxima λmax are given in nanometer [nm] with relative intensities in [%].

2. Synthetic Procedure

5,8-dibromo-2-hexyl-1H-benzo[de]isoquinoline-1,3(2H)-dione (1a)

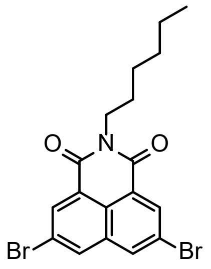

Chemical Formula: $C_{18}H_{17}Br_2NO_2$
Exact Mass: 436.96260

5,8-dibromo-1,8-naphthalenedicarboxylic anhydride was synthesized as reported[1]. In a 250 mL round-bottom flask with a magnetic stirring bar, 3.0 g (8.5 mmol) of 5,8-dibromo-1,8-naphthalenedicarboxylic anhydride was dissolved in 150 mL of Ethanol. To this mixture, 1.7 mL (13 mmol) of n-hexyl amine was added. The reaction mixture was stirred at 80°C for 20 hours. After cooling to room temperature, the precipitate was collected by filtration, washed with 50 mL of cold Ethanol, and dried under vacuum. Yellow solids were obtained with 84% yield.

^{1}H NMR [400 MHz, $CDCl_3$] δ 8.63 (d, J = 1.8 Hz, 2H), 8.26 (d, J = 1.7 Hz, 2H), 4.16 – 4.12 (m, 2H), 1.70 (t, J = 7.6 Hz, 2H), 1.42 – 1.37 (m, 2H), 1.35 – 1.31 (m, 4H), 0.91 – 0.87 (m, 3H).

^{13}C NMR [101 MHz, $CDCl_3$] δ 162.66, 134.37, 134.34, 134.08, 125.26, 124.53, 122.46, 77.16, 40.99, 31.65, 28.11, 26.85, 22.69, 14.19.

HRMS (APPI, DCM): Chemical Formula: $C_{18}H_{17}Br_2NO_2$ cal. 436.962, found 438.961 $[M+2]^+$.

5-bromo-2-hexyl-1H-benzo[de]isoquinoline-1,3(2H)-dione (1b)

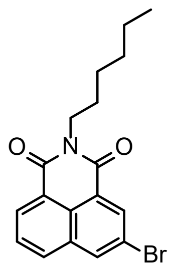

Chemical Formula: $C_{18}H_{18}BrNO_2$
Exact Mass: 359.05209

5-bromo-1,8-naphthalenedicarboxylic anhydride was synthesized as reported[5]. In a 250 mL round-bottom flask, 1.20 g (4.4 mmol) of 5-dibromo-1,8-naphthalenedicarboxylic anhydride was dissolved in 100 mL of Ethanol. To this mixture, 0.63 mL (4.8 mmol) of n-hexyl amine was added. The reaction mixture was stirred at 80°C for 12 hours. After cooling to room temperature, the precipitate was collected by filtration, washed with 25 mL of cold Ethanol, and dried under vacuum. Yellow solids were obtained with 80% yield.

^{1}H NMR [400 MHz, $CDCl_3$] δ 8.66 (d, J = 1.9 Hz, 1H), 8.60 (dd, J = 7.3, 1.2 Hz, 1H), 8.36 (d, J = 1.9 Hz, 1H), 8.12 (dd, J = 8.3, 1.2 Hz, 1H), 7.78 (dd, J = 8.3, 7.3 Hz, 1H), 4.19 – 4.12 (m, 2H), 1.75 – 1.68 (m, 2H), 1.41 (d, J = 7.6 Hz, 2H), 1.34 (dt, J = 7.3, 3.1 Hz, 4H), 0.89 (td, J = 5.9, 4.9, 2.3 Hz, 3H).

^{13}C NMR [101 MHz, $CDCl_3$] δ 163.81, 163.18, 135.54, 134.13, 132.99, 132.84, 131.47, 128.16, 126.78, 124.47, 123.10, 121.25, 40.82, 31.68, 28.17, 26.90, 22.70, 14.20.

HRMS (ESI, DCM): Chemical Formula: $C_{18}H_{18}BrNO_2$ cal. 359.052, found 360.0582 $[M+1]^+$.

2-hexyl-5,8-bis(4,4,5,5-tetramethyl-1,3,2-dioxaborolan-2-yl)-1H-benzo[de]isoquinoline-1,3(2H)-dione (2a)

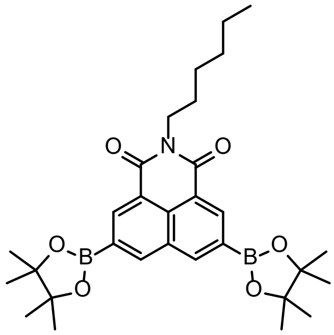

Chemical Formula: $C_{30}H_{41}B_2NO_6$
Exact Mass: 533.31200

In a 250 mL flask with a magnetic stir bar, 1.0 g (2.3 mmol) of **1a**, 2.31 g (9.2 mmol) of bis(pinacolato)diboron, 2.23 g (23 mmol) of potassium acetate and 335 mg (0.45mmol) Pd(dppf)Cl_2 were dissolved in 120 mL of Dioxane. The mixture was heated at 100°C for 24 hours. After cooling, the solvent was removed under vacuum. The crude product to this crude mixture 80 mL of water was added. The aqueous layer was extracted with DCM (2x60mL) and the combined layers were dried over Na_2SO_4. The solvent was removed under reduced pressure. The compound was purified using silica gel chromatography in (1:5) EtOAc:iso-Hexane. Light-green, yellow solids, yield 66%.

^{1}H NMR [400 MHz, $CDCl_3$] δ 8.90 – 8.87 (m, 2H), 8.62 – 8.59 (m, 2H), 4.09 (d, J = 7.8 Hz, 2H), 1.66 – 1.61 (m, 2H), 1.31 (s, 24H), 1.27 – 1.22 (m, 6H), 0.80 (s, 3H).

^{13}C NMR [101 MHz, $CDCl_3$] δ 162.66, 134.37, 134.34, 134.08, 125.26, 124.53, 122.46, 77.16, 40.99, 31.65, 28.11, 26.85, 22.69, 14.19.

HRMS (APPI, DCM): Chemical Formula: $C_{30}H_{41}B_2NO_6$ cal. 533.312, found 533.299 $[M+2H]^+$.

2-hexyl-5-(4,4,5,5-tetramethyl-1,3,2-dioxaborolan-2-yl)-1H-benzo[de]isoquinoline-1,3(2H)-dione (2b)

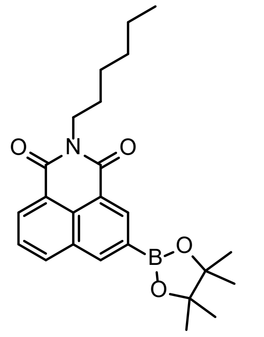

Chemical Formula: $C_{24}H_{30}BNO_4$
Exact Mass: 407.22679

In a 100 mL round-bottom flask, 1.0 g (2.8 mmol) of **1b**, 1.41 g (5.5 mmol) of bis(pinacolato)diboron, 1.4 g (14 mmol) of potassium acetate and 200 mg (0.28mmol) Pd(dppf)Cl_2 were dissolved in 80 mL of dioxane. The mixture was heated at 100°C for 24 hours. After cooling, the solvent was removed under vacuum. The crude product to this crude mixture 30 mL of water was added. The aqueous layer was extracted with DCM (2x40mL) and the combined layers were dried over Na_2SO_4. The solvent was removed under reduced pressure. The compound was purified using silica gel chromatography in (1:5) EtOAc:iso-Hexane. white solids, yield 72%.

^{1}H NMR [400 MHz, $CDCl_3$] δ 8.98 (d, J = 1.1 Hz, 1H), 8.67 (d, J = 1.1 Hz, 1H), 8.62 (dd, J = 7.3, 1.2 Hz, 1H), 8.23 (dd, J = 8.2, 1.2 Hz, 1H), 7.74 (dd, J = 8.2, 7.3 Hz, 1H), 4.21 – 4.15 (m, 2H), 1.72 (ddd, J = 9.6, 4.5, 2.2 Hz, 2H), 1.44 – 1.46 (m, 2H)1.41 (s, 12H), 1.33 (tt, J = 5.7, 2.6 Hz, 4H), 0.91 – 0.86 (m, 3H).

^{13}C NMR [101 MHz, $CDCl_3$] δ 164.38, 141.53, 136.51, 134.58, 132.08, 131.17, 129.66, 126.99, 122.93, 122.06, 84.71, 40.57, 31.73, 28.22, 26.93, 25.09, 22.71, 14.22.

HRMS (APPI, DCM): Chemical Formula: $C_{24}H_{30}BNO_4$ cal. 407.226, found 408.2358 $[M+H]^+$.

6-[2-(2-Chlorophenyl)ethenyl]-1,4-difluorobenzo[c]phenanthrene (3b)

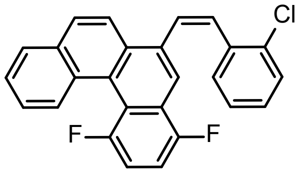

Chemical Formula: $C_{26}H_{15}ClF_2$
Exact Mass: 400.08303

The compound was synthesized by modifying procedure[2]. In a 250 mL flask equipped with a magnetic stir bar, 1.85 g (3 mmol) of [(1,4-Difluorobenzo[c]phenanthren-6-yl)methyl]triphenylphosphonium were dissolved in HPLC-grade dichloromethane. The atmosphere was evacuated and replaced with argon. The flask was cooled in an ice-water bath. After temperature equilibration, 4.98 g (124.4 mmol) of NaOH dissolved in 5 mL H_2O were added, and the flask was degassed. After 30 minutes, the mixture turned orange red. Afterwards, 350 mg (2.5 mmol) of 2-chlorobenzaldehyde was added under an argon flow. The atmosphere was evacuated and replaced with argon. The reaction mixture was stirred at ambient temperature for 12 hours. The mixture was then quenched with 20 mL of water, and the aqueous layer was extracted with DCM (2 × 30 mL). The compound was purified using silica gel chromatography (1:5 DCM:hexane). Yield 80% (800 mg, 2 mmol).

^{1}H NMR [400 MHz, $CDCl_3$] δ 8.32 – 8.22 (m, 1H), 8.12 (d, J = 8.8 Hz, 1H), 8.05 – 7.96 (m, 2H), 7.88 (dd, J = 2.0, 1.1 Hz, 1H), 7.67 – 7.59 (m, 2H), 7.37 (dd, J = 8.0, 1.2 Hz, 1H), 7.28 – 7.25 (m, 1H), 7.23 – 7.16 (m, 2H), 7.03 (td, J = 7.7, 1.7 Hz, 1H), 6.86 (dd, J = 7.8, 1.7 Hz, 1H), 6.71 (td, J = 7.6, 1.2 Hz, 1H).

^{13}C NMR [101 MHz, $CDCl_3$] δ 156.28, 156.01, 153.77, 153.55, 134.78, 134.50, 133.85, 132.68, 130.68, 130.45, 130.40, 129.88, 129.71, 129.62, 129.45, 129.01, 128.57, 127.26, 126.41, 126.22, 125.05, 122.39, 119.53, 119.49, 111.98, 111.89, 111.70, 111.62, 110.80, 110.70, 110.57, 110.47.

^{19}F NMR [473 MHz, $CDCl_3$] δ -103.61-103.73(m), -126.83, -126.84 - 126.91(m).

4-Chloro-13,16-difluorobenzo[s]picene (4a)

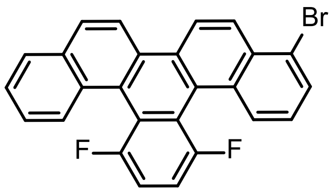

Chemical Formula: $C_{26}H_{13}BrF_2$
Exact Mass: 442.01687

Compound was obtained according to the reported procedure[3] as a white solid (yield 81%).

4-Chloro-13,16-difluorobenzo[s]picene (4b)

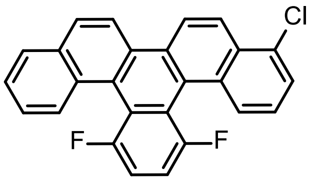

Chemical Formula: $C_{26}H_{13}ClF_2$
Exact Mass: 398.06738

The compound was synthesized by modifying Procedure[2]. In a 500 mL flask with a magnetic stir bar, 810 mg (2 mmol) of **3** and 400 mL of cyclohexane were combined. Then, 1.2 g (2.42 mmol) of I_2 were added, and the mixture was vigorously stirred. The reaction mixture was irradiated in photoreactor using a 400 W high-pressure mercury-vapor lamp under ambient conditions and air. Upon full conversion, the mixture was quenched with $Na_2S_2O_3$, and the aqueous layer was extracted with DCM (2 × 60 mL). The combined organic layers were dried over anhydrous $MgSO_4$, filtered, and concentrated. Afterwards, 10 mL of MeOH were added, precipitating the product to yield 613 mg (1.54 mmol, 77%).

^{1}H NMR [400 MHz, $CDCl_3$] δ 8.64 (d, J = 9.2 Hz, 1H), 8.61 – 8.51 (m, 2H), 8.27 – 8.18 (m, 1H), 8.18 – 8.08 (m, 2H), 8.01 – 7.94 (m, 1H), 7.66 (d, J = 6.8 Hz, 1H), 7.64 – 7.57 (m, 2H), 7.49 (t, J = 8.0 Hz, 1H), 7.43 – 7.34 (m, 2H).

^{13}C NMR [101 MHz, $CDCl_3$] δ 132.48, 131.27, 130.40, 130.15, 130.10, 129.74, 129.71, 129.58, 128.87, 128.73, 127.18, 126.36, 125.39, 125.37, 125.34, 125.01, 124.99, 120.98, 119.63, 114.86, 114.76, 114.63, 114.58, 114.53, 114.48, 114.36, 114.26, 77.31, 76.99, 76.67.

1-Chloro-as-indaceno[3,2,1,8,7,6-pqrstuv]picene (5a)

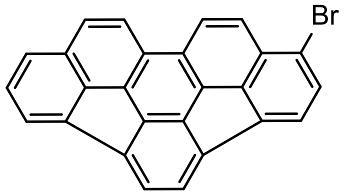

Chemical Formula: $C_{26}H_{11}Br$
Exact Mass: 402.00441
Molecular Weight: 403.27800

The compound was synthesized without any change in the procedure[3] as a yellow solid, yield 65%.

1-Chloro-as-indaceno[3,2,1,8,7,6-pqrstuv]picene (5b)

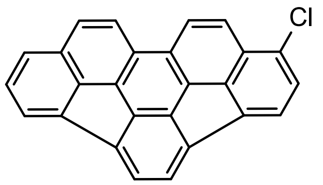

Chemical Formula: $C_{26}H_{11}Cl$
Exact Mass: 358.05493
Molecular Weight: 358.82400

In a glass tube, 5 grams of aluminium oxide (Aluminium oxide 90 active neutral, Millipore) was heated to 250°C under a vacuum of 0.02 Bar for 15 min. The temperature was then raised to 550°C for an additional 30 minutes at the same pressure. After cooling to room temperature, the **4b** was added under an argon flow, and the tube was evacuated to 1.5×10^{-2} bar and sealed. The sealed ampule was heated at 250°C for 5 h. The reaction mixture was extracted using Soxhlet extraction with toluene. Yellow solid (yield 65%).

^{1}H NMR [400 MHz, $CDCl_3$] δ 8.15 (dd, J = 19.6, 8.8 Hz, 2H), 7.97 (d, J = 8.8 Hz, 1H), 7.82 (d, J = 8.8 Hz, 1H), 7.73 (d, J = 6.9 Hz, 1H), 7.69 – 7.63 (m, 3H), 7.60 (d, J = 11.7 Hz, 1H), 7.42 (dd, J = 8.1, 7.0 Hz, 1H), 7.37 (d, J = 7.4 Hz, 1H).

^{13}C NMR [126 MHz, $C_2D_2Cl_4$] δ 138.76, 138.59, 138.22, 137.82, 137.69, 137.62, 137.57, 136.35, 131.87, 130.17, 129.40, 129.06, 129.04, 128.11, 127.10, 126.95, 126.88, 125.88, 125.67, 124.86, 124.05, 123.90, 123.75, 120.18.

MS (LDI) m/z (rel.int.): 358.0556 $[M]^+$

HRMS (APPI, DCM): Chemical Formula: $C_{26}H_{11}Cl$ cal. 358.0549, found 359.0638$[M+H]^+$.

UV/Vis (1,1,2,2-tetrachloroethane; 313 K): λ [nm]) = 280-380nm.

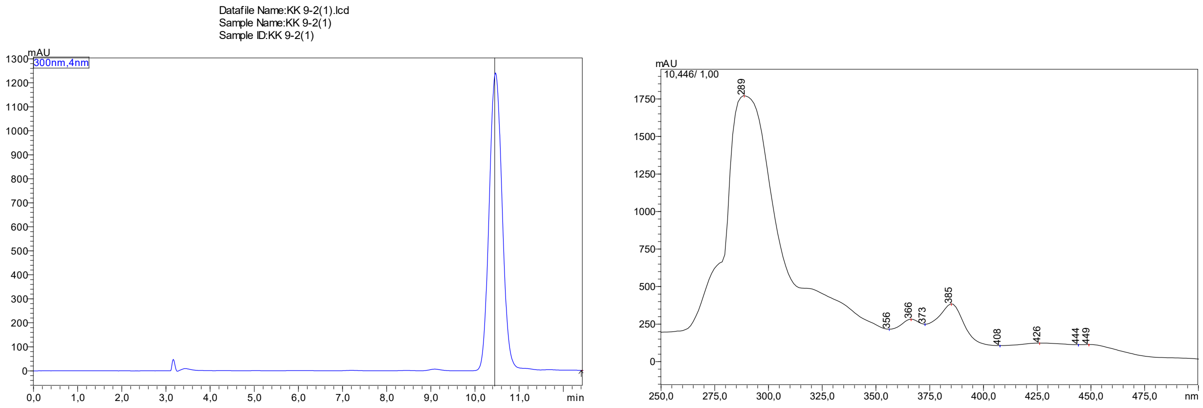

*Figure S 1. HPLC profile and UV-vis spectrum of compound **5b**. Conditions: Tol:MeOH 4:6, PBr column, flow rate 1 ml/min.*

as-indaceno[3,2,1,8,7,6-pqrstuv]picene (6)

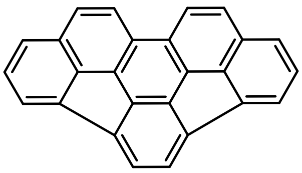

Chemical Formula: $C_{26}H_{12}$
Exact Mass: 324.09390
Molecular Weight: 324.38200

The compound was synthesized according to the reported procedure[4].

5,8-bis(as-indaceno[3,2,1,8,7,6-pqrstuv]picen-1-yl)-2-hexyl-1H-benzo[de]isoquinoline-1,3(2H)-dione (7)

Route I from 5b

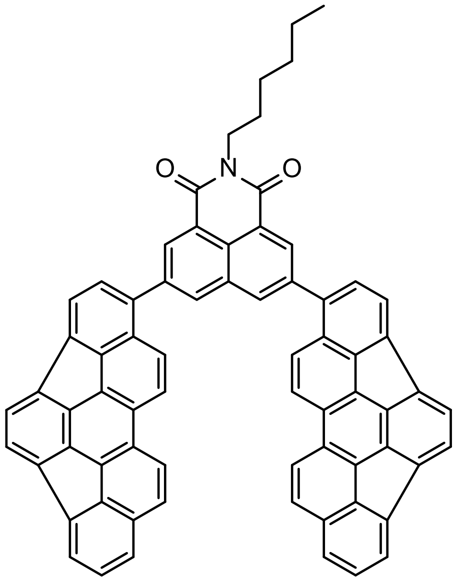

Chemical Formula: $C_{70}H_{39}NO_2$
Exact Mass: 925.29808

To a MW tube containing 5 mL of toluene, 26 µmol of **2a** and 55 µmol of **5b** were added. The mixture was heated with a heat gun until fully dissolved. Then, 2 mL of methanol and 261 µmol of K_2CO_3 were added, and the solution was slightly heated to help dissolve the base. The MW tube was thoroughly flushed with argon. Under argon flow, 5 µmol of XPhos and 5 µmol of $Pd_2(dba)_3$ were added. The tube was sealed with a cap and placed in a microwave reactor at 110°C for 16 hours. The reaction mixture was filtered through a silica plug using toluene. It was then washed with hot toluene and collected in another flask. Both solutions were analyzed by HPLC (PBr column, 1 mL/min, toluene/methanol = 7/3). The first flask contained a complex mixture of products, while the second flask had an almost pure product. The product from the first flask was further purified by semipreparative HPLC (PBr column, 5 mL/min, DCM/toluene/methanol = 30/42/28). Yield 20%.

^{1}H NMR [600 MHz, $C_2D_2Cl_4$] δ 8.98 (s, 2H), 8.60 (s, 2H), 8.22 (d, J = 8.8 Hz, 2H), 8.18 (d, J = 8.7 Hz, 2H), 7.99 (d, J = 8.8 Hz, 2H), 7.92 (d, J = 7.1 Hz, 2H), 7.87 (d, J = 8.7 Hz, 2H), 7.82 – 7.77 (m, 6H), 7.72 (d, J = 8.1 Hz, 2H), 7.66 (d, J = 7.1 Hz, 2H), 7.50 – 7.46 (m, 2H), 4.31 – 4.25 (m, 2H), 1.53 (d, J = 7.2 Hz, 2H), 1.43 (d, J = 6.1 Hz, 6H), 0.96 (d, J = 7.1 Hz, 3H).

^{13}C NMR [151 MHz, $C_2D_2Cl_4$] δ 164.05, 139.51, 139.15, 139.01, 138.89, 138.52, 138.21, 138.07, 137.47, 136.74, 134.20, 132.81, 130.16, 129.97, 129.78, 129.32, 129.14, 127.21, 127.14, 127.02, 125.94, 125.88, 125.43, 125.11, 124.08, 123.84, 123.84, 123.68, 123.25, 120.38, 99.62, 31.59, 29.70, 26.90, 22.69, 22.63, 14.16.

MS (LDI) m/z (rel.int.): 925.2986 $[M]^+$

HRMS (APPI, DCM): Chemical Formula: $C_{70}H_{39}NO_2$ cal. 925.2980, found 926.3000 $[M+H]^+$.

UV/Vis (1,1,2,2-tetrachloroethane; 313 K): λ [nm]) = 300-400nm. ε = 37800 $M^{-1}cm^{-1}$ (297 nm)

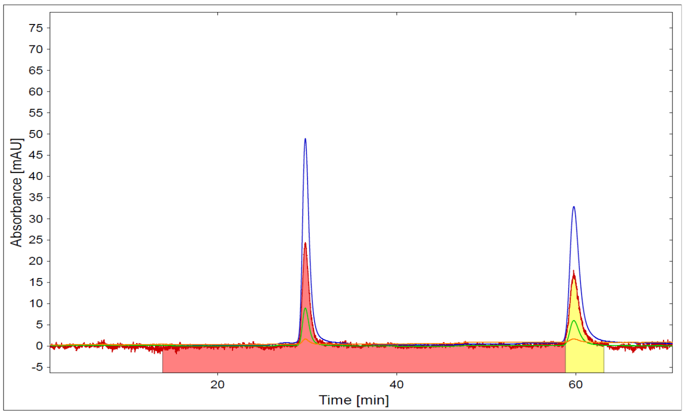

Marker table

	Color	Marker	From [min]	To [min]	Comment
1		Recycle-1	13.884	58.849	Recycle-1
2		Fraction-1	58.849	63.137	Collection-1

Figure S 2. HPLC GPC (2 cycles) Chromatogram spectrum in toluene of tweezer 7.

Route II from 5a

In a 25 mL round-bottom flask 26 µmol of **2a** dissolved in 8 mL of toluene. To this solution, 55 µmol of **5a** were added. 131 µmol of K_2CO_3 were dissolved in 0.2 mL of H_2O and added to the flask. The atmosphere was replaced with argon. Under argon flow, 6.5 µmol of $Pd(PPh_3)_4$ were introduced. The reaction mixture was then subjected to reflux for 16 hours. After completion, the mixture was filtered through a silica plug using toluene and analyzed by HPLC (PBr column, 1 mL/min, toluene/methanol = 7/3). Only trace amounts of the product were detected.

Route III from 5a

46 µmol of **5a** were placed into a one-neck round-bottom flask and dissolved in a mixture of 5 mL toluene and 5 mL DMSO. To this solution, 19 µmol of **2a** and 90 µmol of K_2CO_3 in 5 mL toluene were added. The atmosphere was replaced with argon. Under argon flow, 1.9 µmol of $Pd(dppf)Cl_2$ were introduced. The reaction mixture was then heated to 80°C for 16 hours. Upon completion, the reaction mixture was quenched with water. The aqueous layer was extracted with toluene, and the combined organic layers were analyzed by HPLC (PBr column, 1 mL/min, toluene/methanol = 7/3). The chromatogram showed mainly the starting material with some traces of the target product.

5-(as-indaceno[3,2,1,8,7,6-pqrstuv]picen-1-yl)-2-hexyl-1H-benzo[de]isoquinoline-1,3(2H)-dione (8)

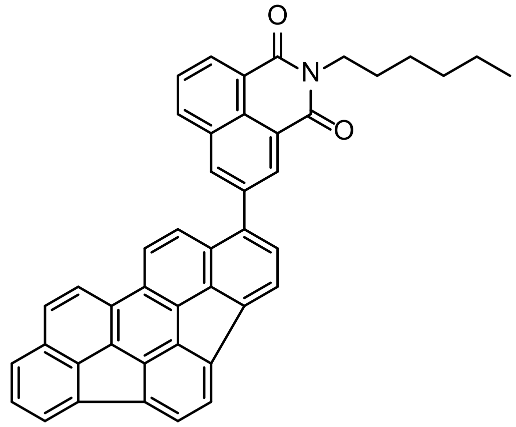

Chemical Formula: $C_{44}H_{29}NO_2$
Exact Mass: 603.21983

To a MW tube containing 5 mL of toluene, 31 µmol of **2b** and 37 µmol of **5b** were added. The mixture was heated with a heat gun until fully dissolved. 2 mL of methanol and 308 µmol of K_2CO_3 were added. The solution was slightly heated to help dissolve the base. The MW tube was thoroughly flushed with argon. Under argon flow, 6 µmol of XPhos and 6 µmol of $Pd_2(dba)_3$ were added. The tube was sealed with a cap and placed in a microwave reactor at 110°C for 16 hours. The reaction mixture was filtered through a silica plug using hot toluene and analyzed by HPLC (PBr column, 1 mL/min, toluene/methanol = 7/3). The product was isolated by semipreparative HPLC (PBr column, 5 mL/min, toluene/methanol = 1/1). Yield 20%.

^{1}H NMR [600 MHz, $C_2D_2Cl_4$] δ 8.83 (d, J = 1.7 Hz, 1H), 8.55 (dd, J = 7.3, 1.1 Hz, 1H), 8.43 – 8.42 (m, 1H), 8.28 – 8.25 (m, 1H), 8.10 (dd, J = 8.8, 7.5 Hz, 2H), 7.86 – 7.81 (m, 2H), 7.79 – 7.76 (m, 2H), 7.73 – 7.69 (m, 3H), 7.64 (d, J = 8.1 Hz, 1H), 7.52 (d, J = 7.1 Hz, 1H), 7.39 (dd, J = 8.1, 7.0 Hz, 1H), 4.12 – 4.08 (m, 2H), 1.69 – 1.64 (m, 2H), 1.39 – 1.35 (m, 2H), 1.29 – 1.25 (m, 4H), 0.83 (t, J = 7.1 Hz, 3H).

^{13}C NMR [151 MHz, $C_2D_2Cl_4$] δ 164.01, 139.06, 138.93, 138.66, 138.37, 138.26, 138.19, 138.11, 137.97, 137.93, 137.18, 136.44, 134.12, 134.11, 132.58, 131.73, 131.19, 130.03, 129.79, 129.68, 129.04, 127.50, 127.23, 127.10, 126.91, 126.88, 125.90, 125.82, 125.37, 124.93, 123.99, 123.78, 123.77, 123.42, 122.91, 122.49, 120.18, 99.36, 40.56, 31.47, 28.01, 26.76, 22.54, 14.10.

MS (LDI) m/z (rel.int.): 603.599 $[M]^+$

HRMS (APPI, DCM): Chemical Formula: $C_{44}H_{29}NO_2$ cal. 603.721, found 603.599$[M]^+$.

UV/Vis (1,1,2,2-tetrachloroethane; 313 K): λ [nm]) = 300-450nm. ε = 26500 $M^{-1}cm^{-1}$ (297 nm)

Fluorescence quantum yield: 10.5%

3. NMR Spectra

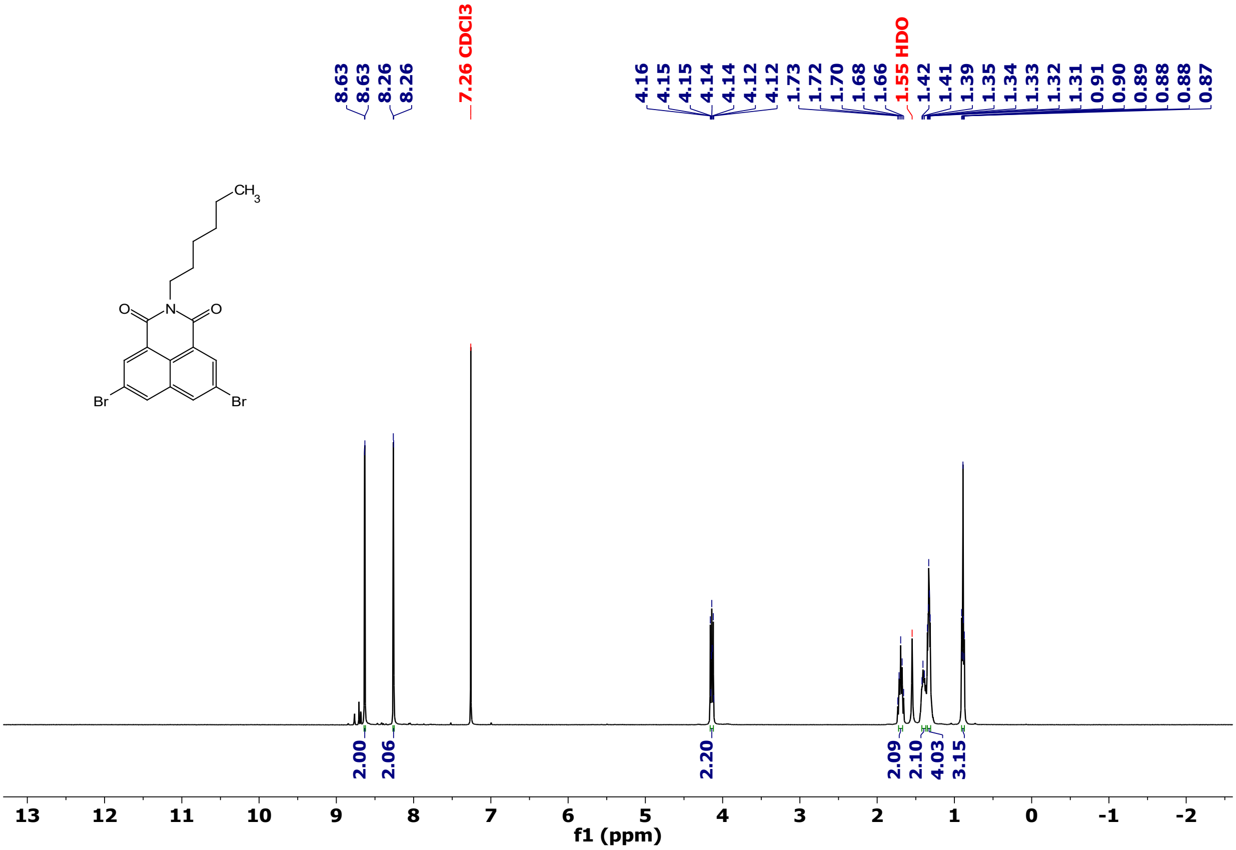

*Figure S 3. ^{1}H NMR (400 MHz, Chloroform-d) spectrum of compound **1a**.*

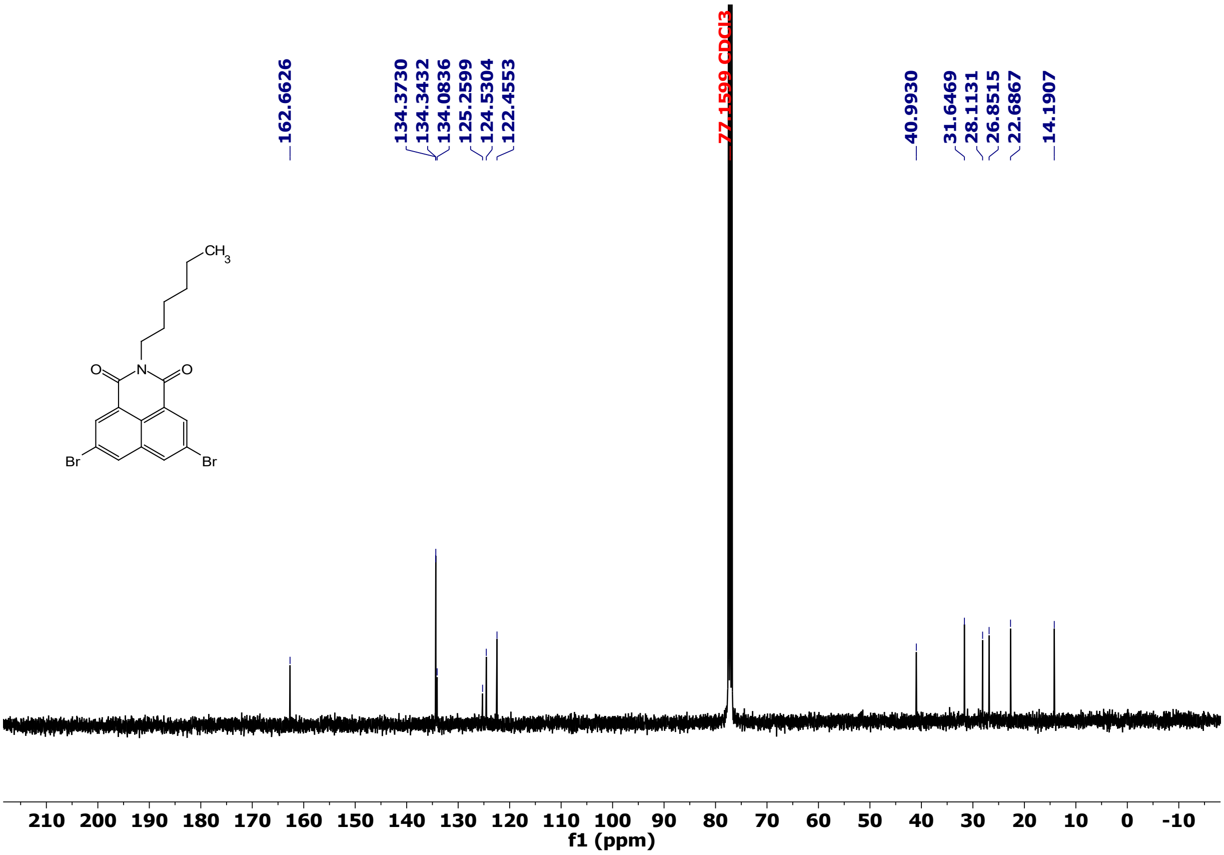

*Figure S 4. ^{13}C NMR (101 MHz, Chloroform-d) spectrum of compound **1a**.*

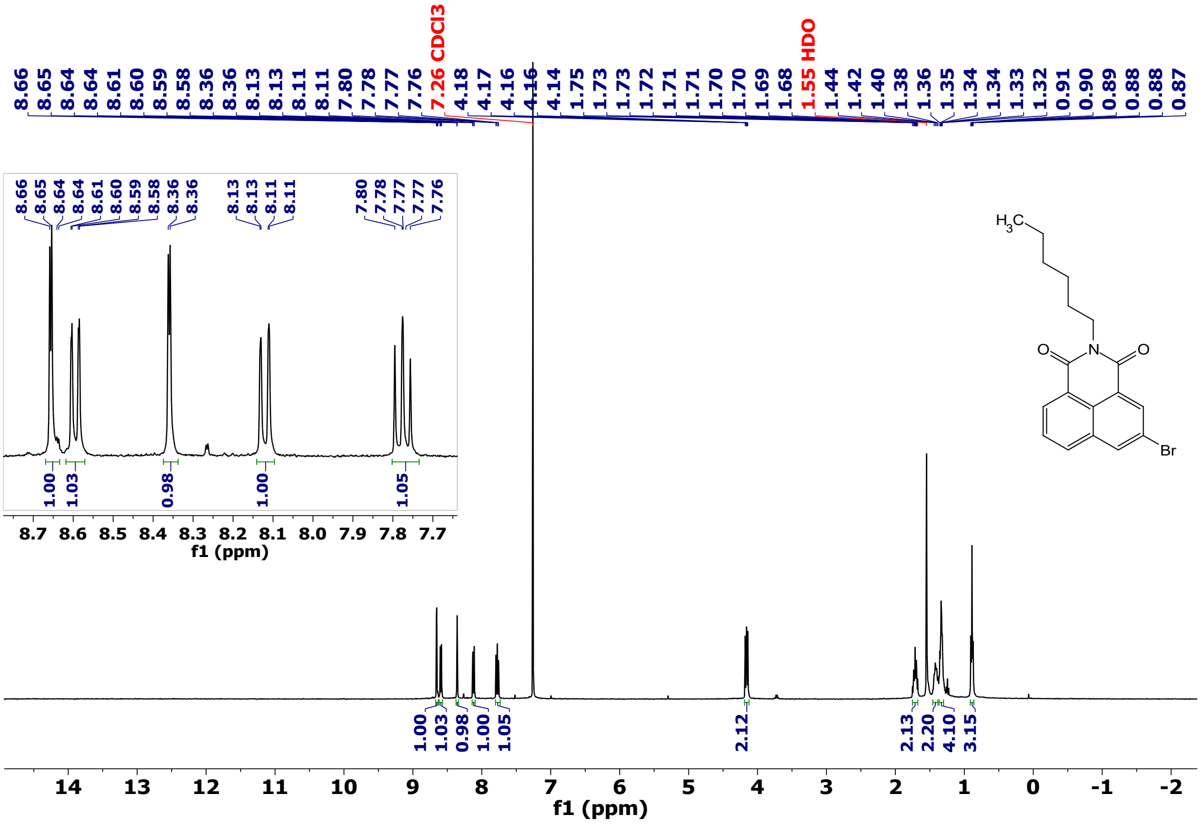

*Figure S 5. ^{13}C NMR (101 MHz, Chloroform-d) spectrum of compound **1b**.*

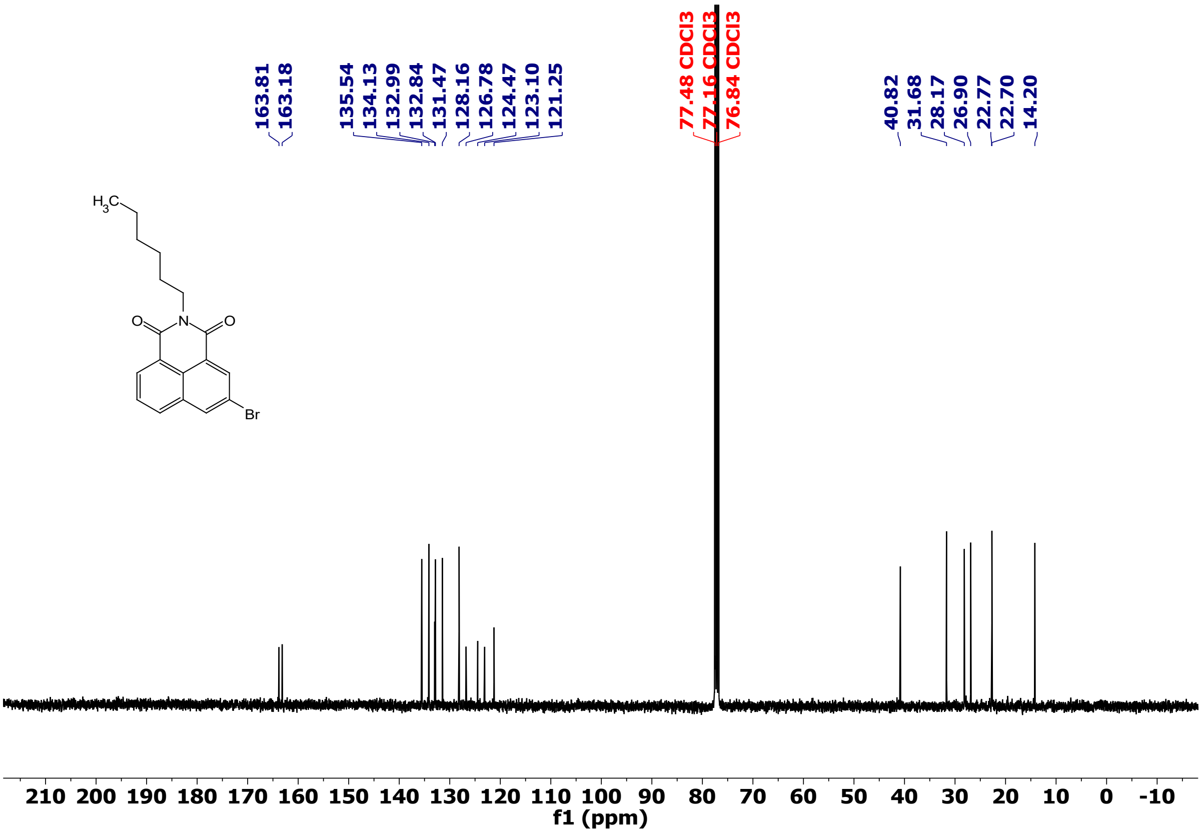

*Figure S 6. ^{13}C NMR (101 MHz, Chloroform-d) spectrum of compound **1b**.*

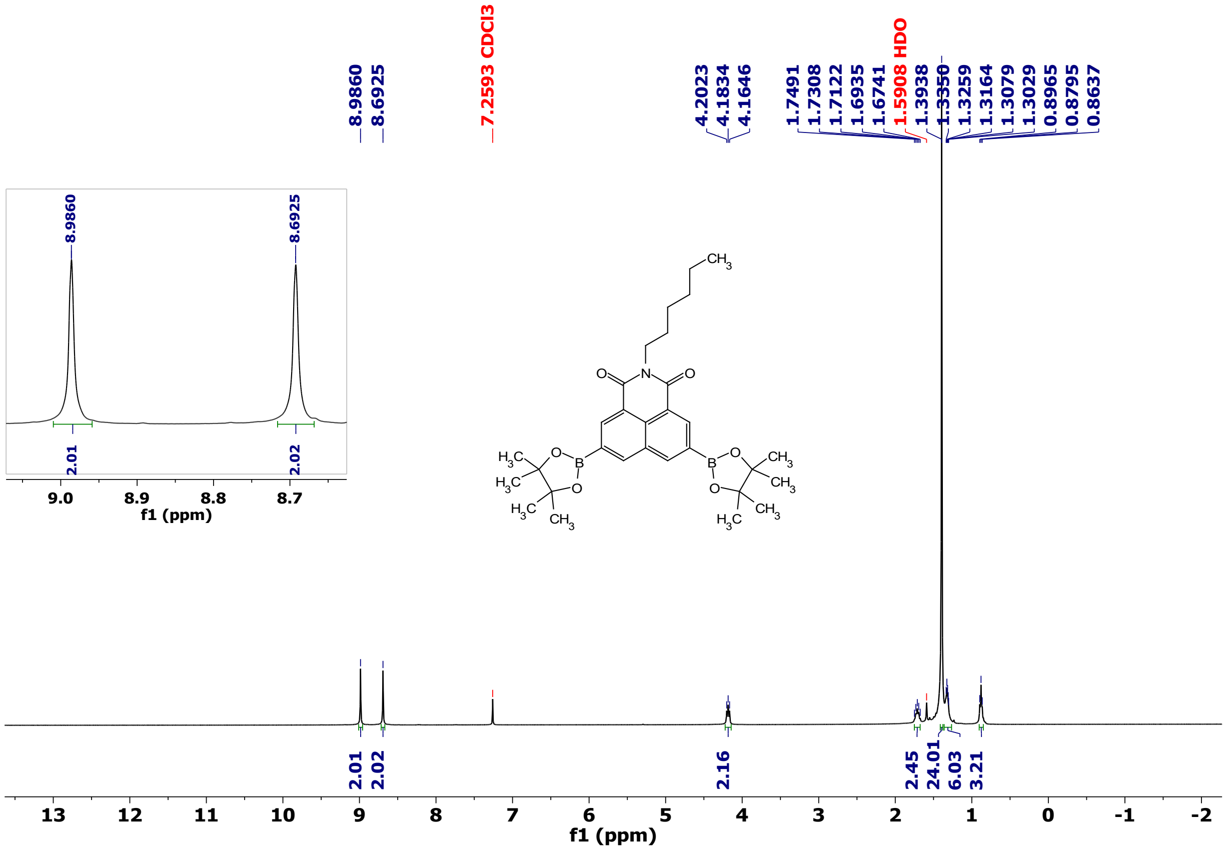

Figure S 7. [13]H NMR (400 MHz, Chloroform-d) spectrum of compound ***2a****.*

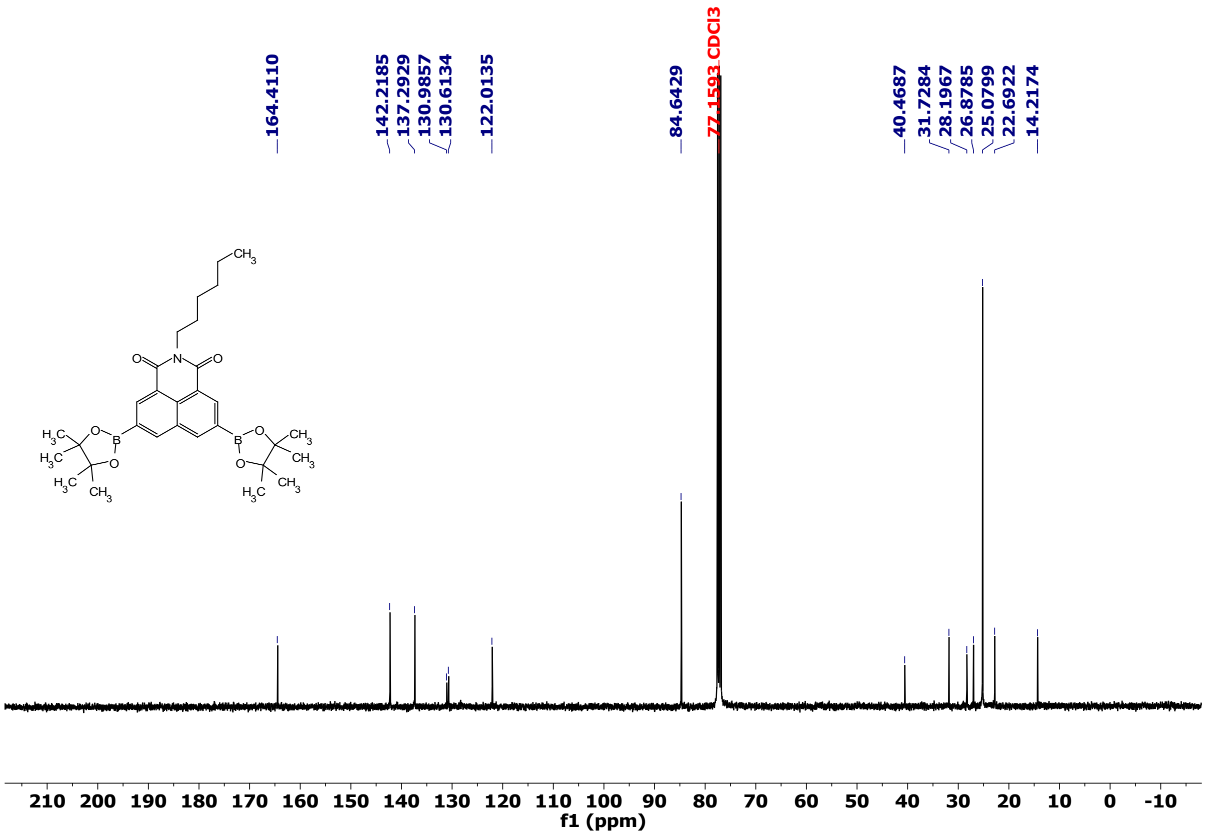

Figure S 8. ^{13}C NMR (101 MHz, Chloroform-d) spectrum of compound ***2a****.*

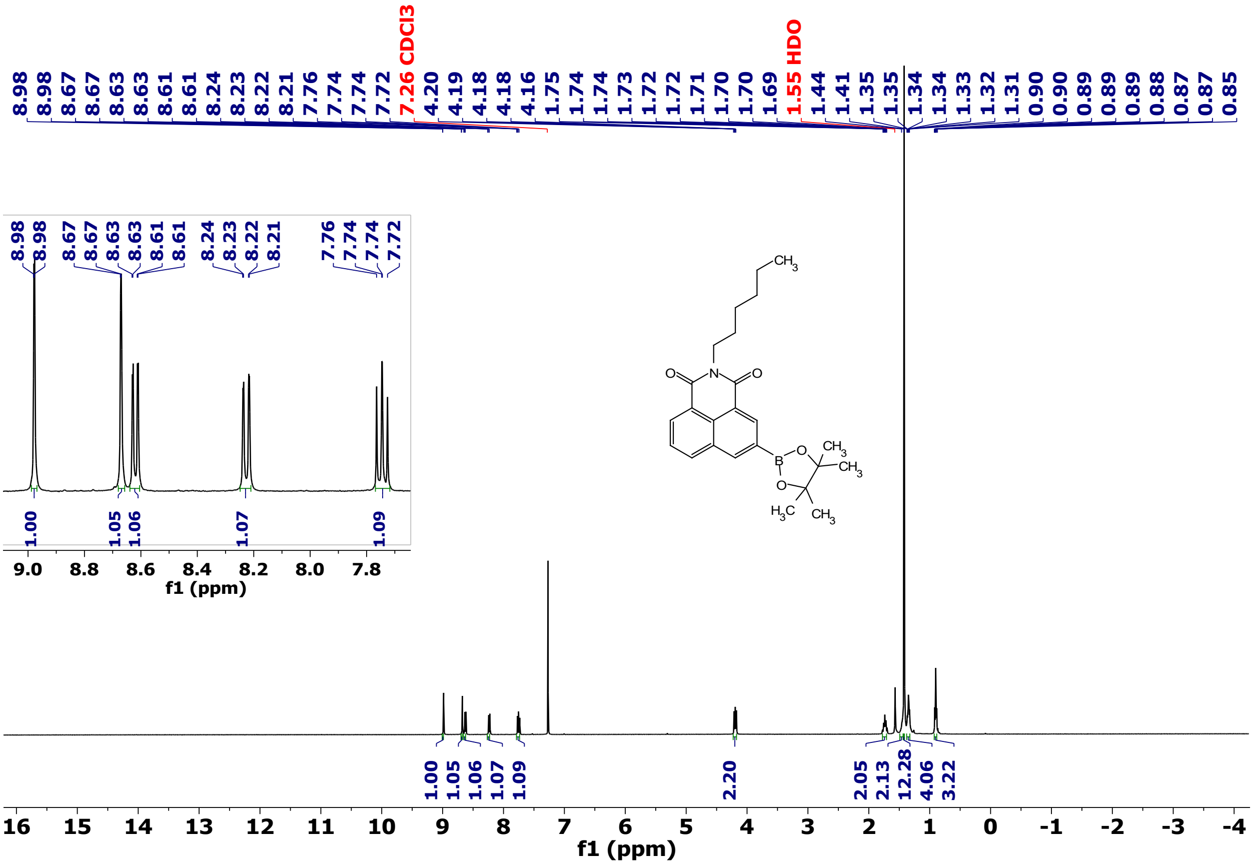

*Figure S 9. ^{13}C NMR (101 MHz, Chloroform-d) spectrum of compound **2b**.*

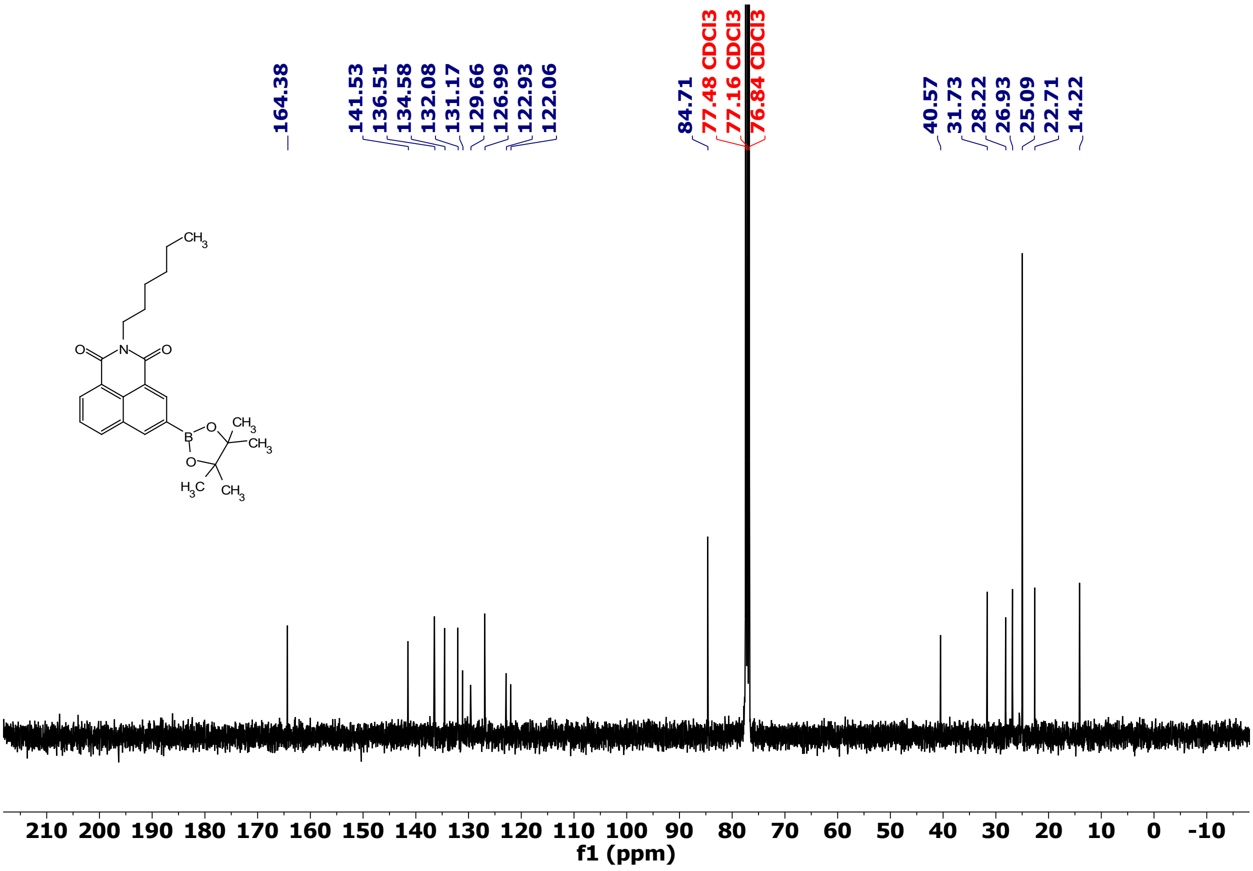

*Figure S 10. ^{13}C NMR (101 MHz, Chloroform-d) spectrum of compound **2b**.*

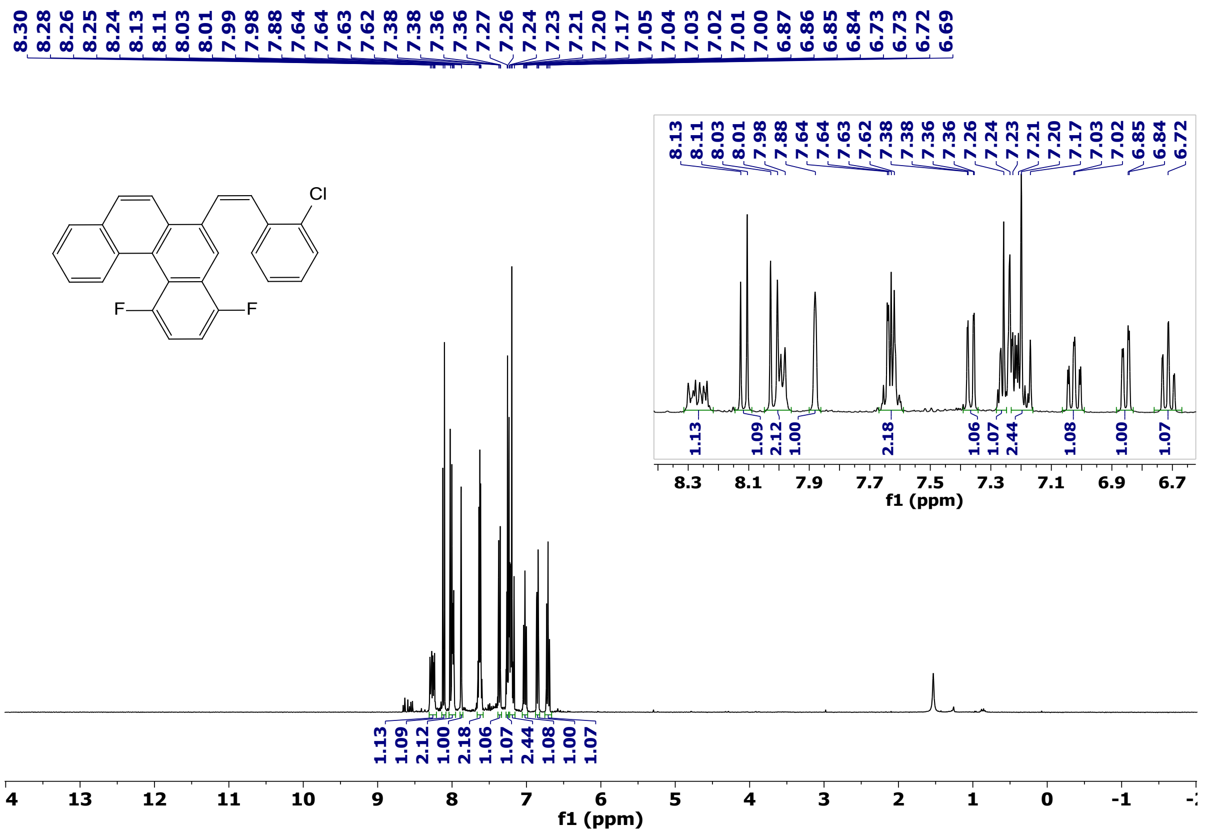

*Figure S 11. ^{1}H NMR (400 MHz, Chloroform-d) spectrum of compound **3b**.*

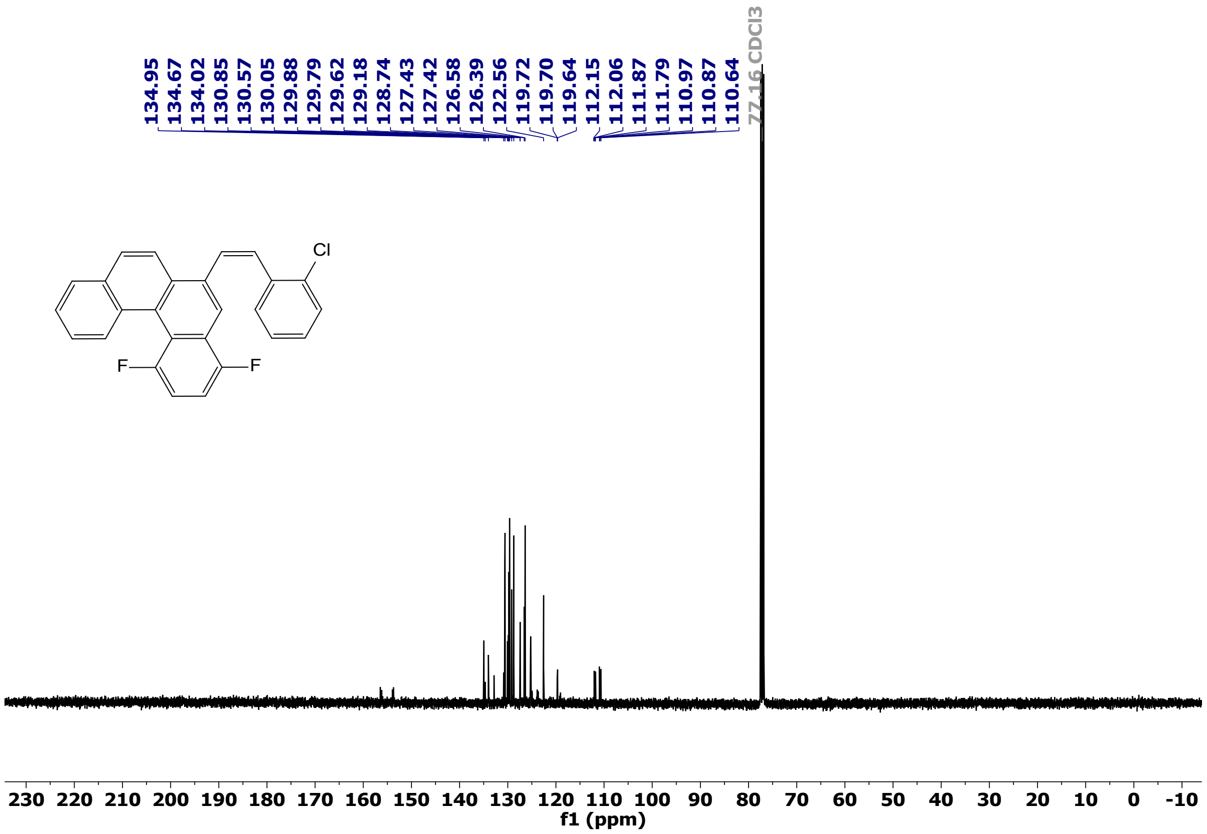

*Figure S 12. ^{13}C NMR (101 MHz, Chloroform-d) spectrum of compound **3b**.*

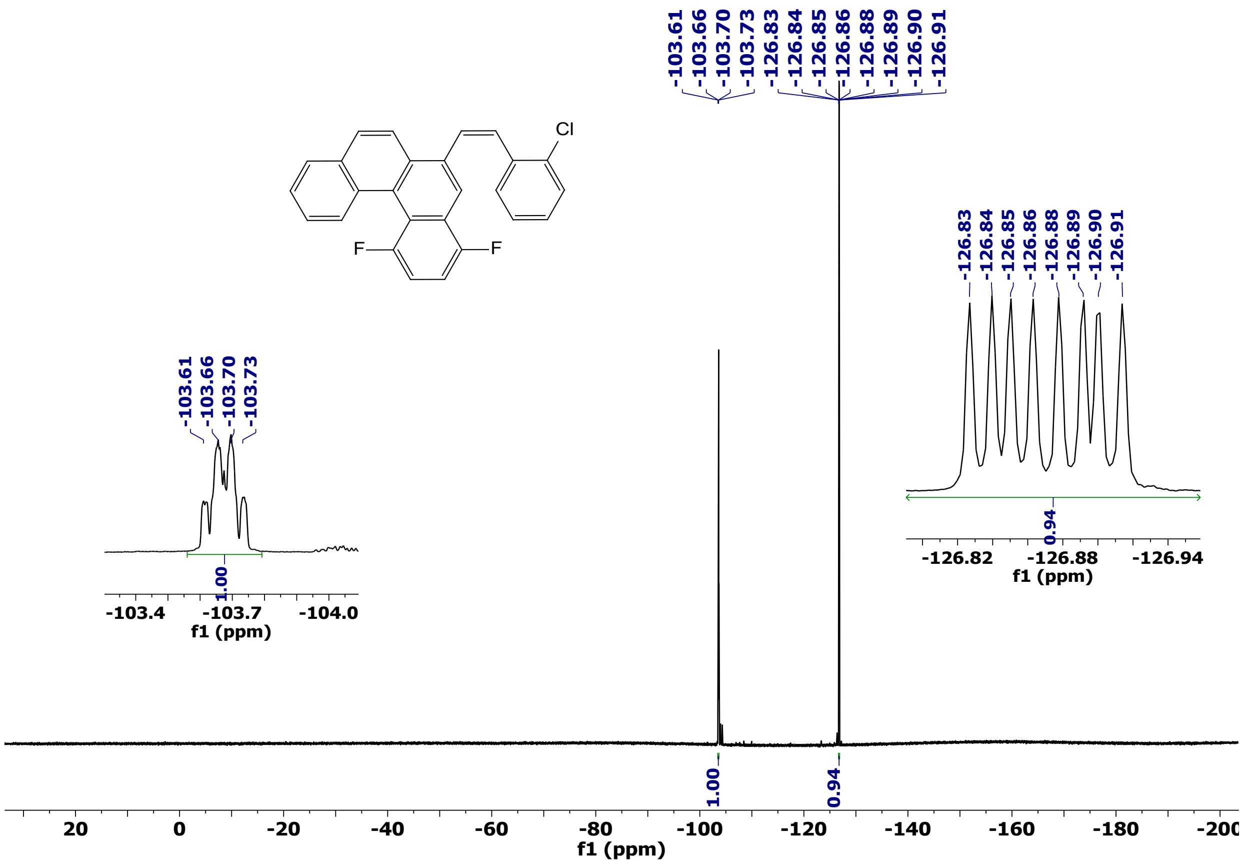

*Figure S 13. ^{19}F NMR (473 MHz, Chloroform-d) spectrum of compound **3b**.*

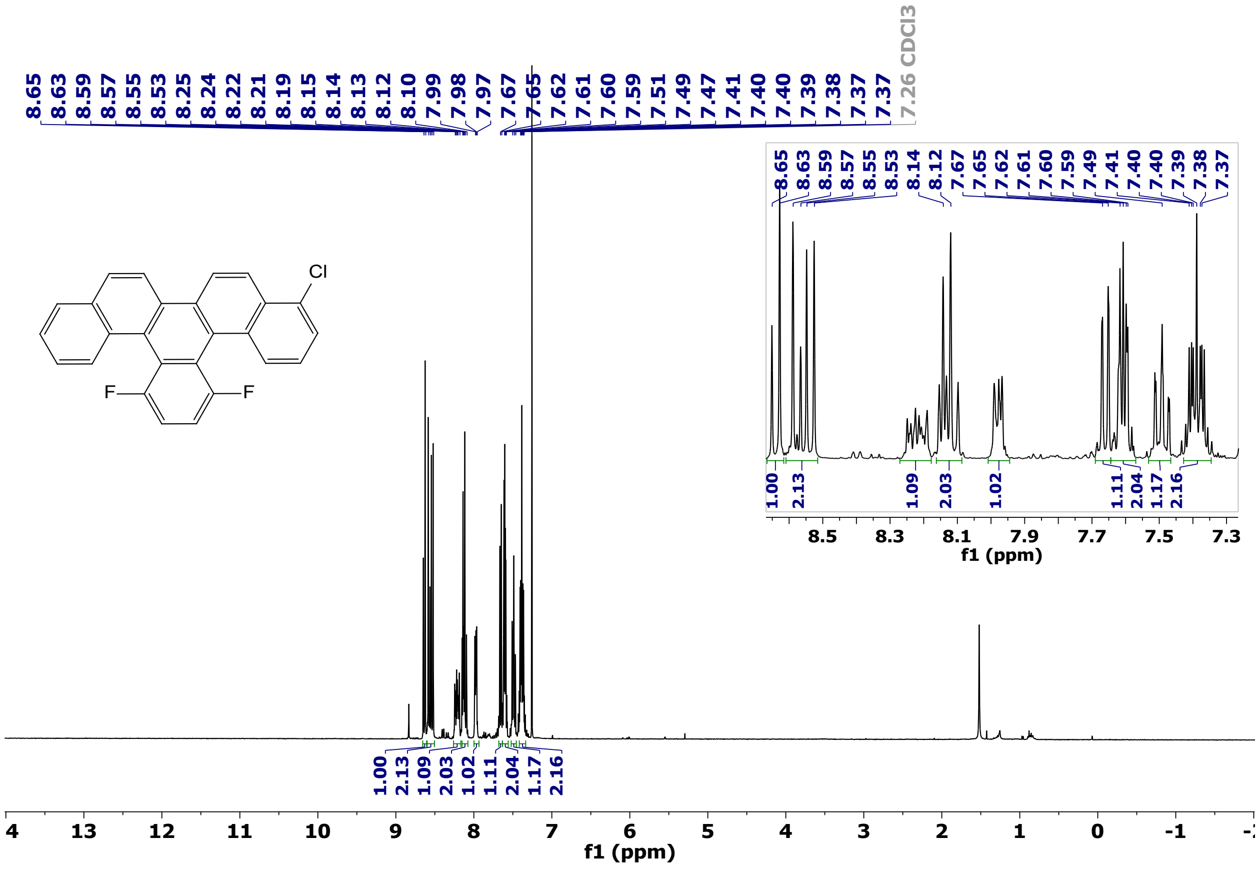

*Figure S 14. ^{1}H NMR (400 MHz, Chloroform-d) spectrum of compound **4b**.*

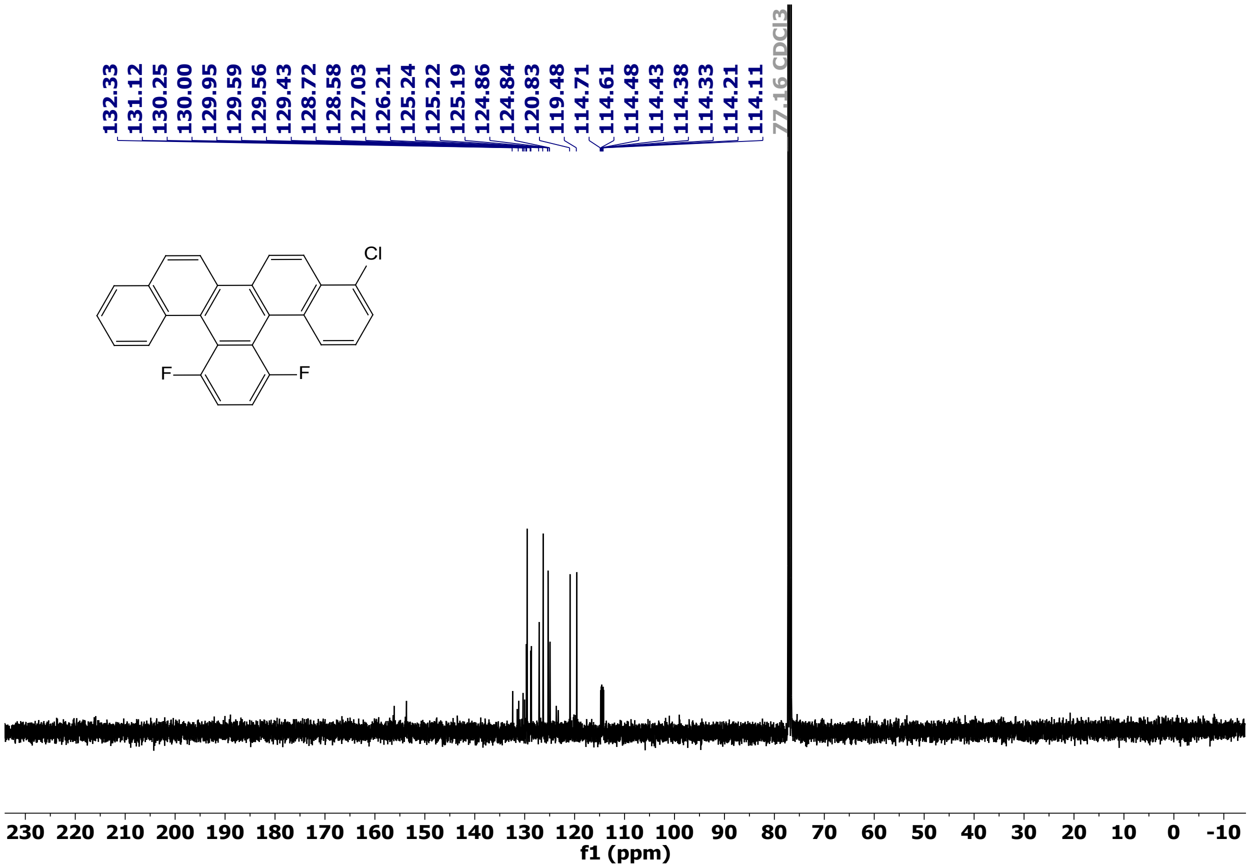

Figure S 15. ^{13}C NMR (101 MHz, Chloroform-d) spectrum of compound ***4b****.*

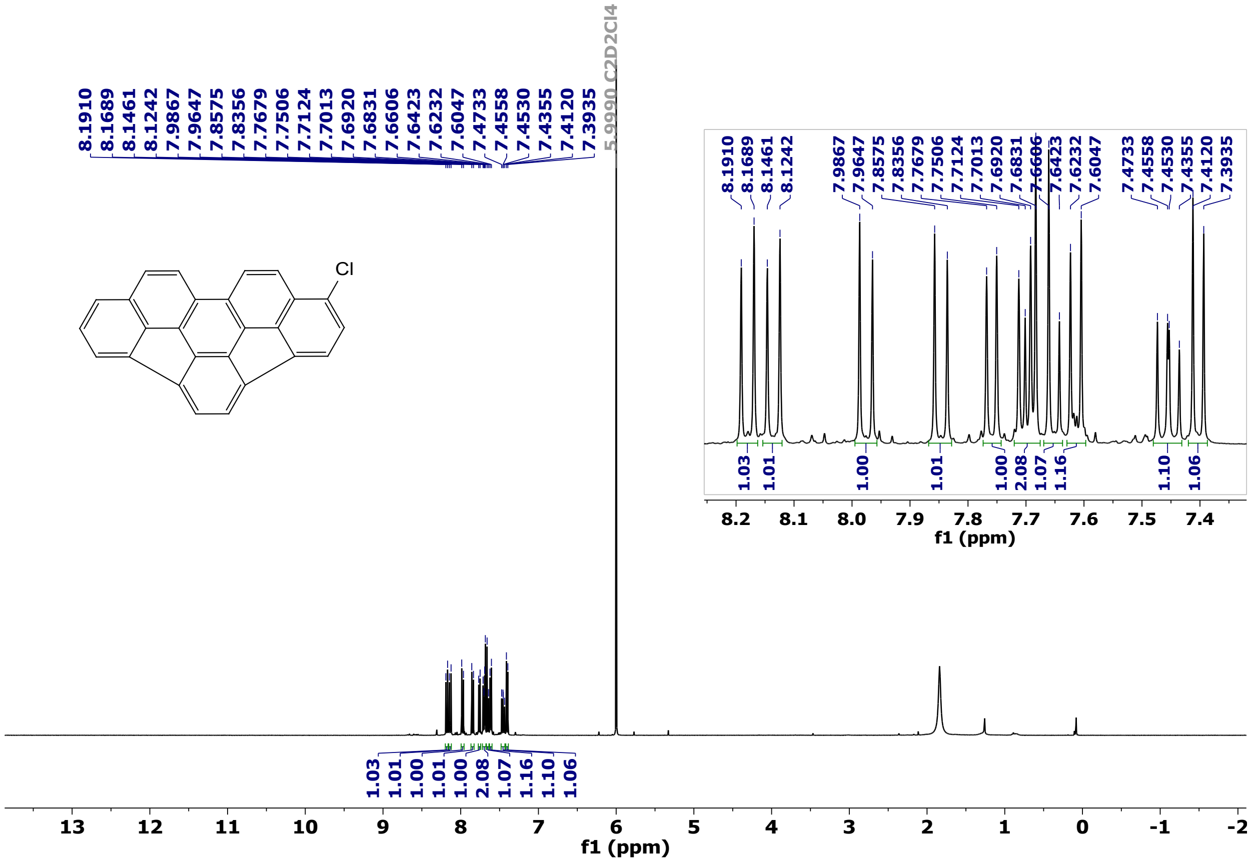

Figure S 16. ^{1}H NMR (400 MHz, $C_2D_2Cl_4$) spectrum of compound ***5b****.*

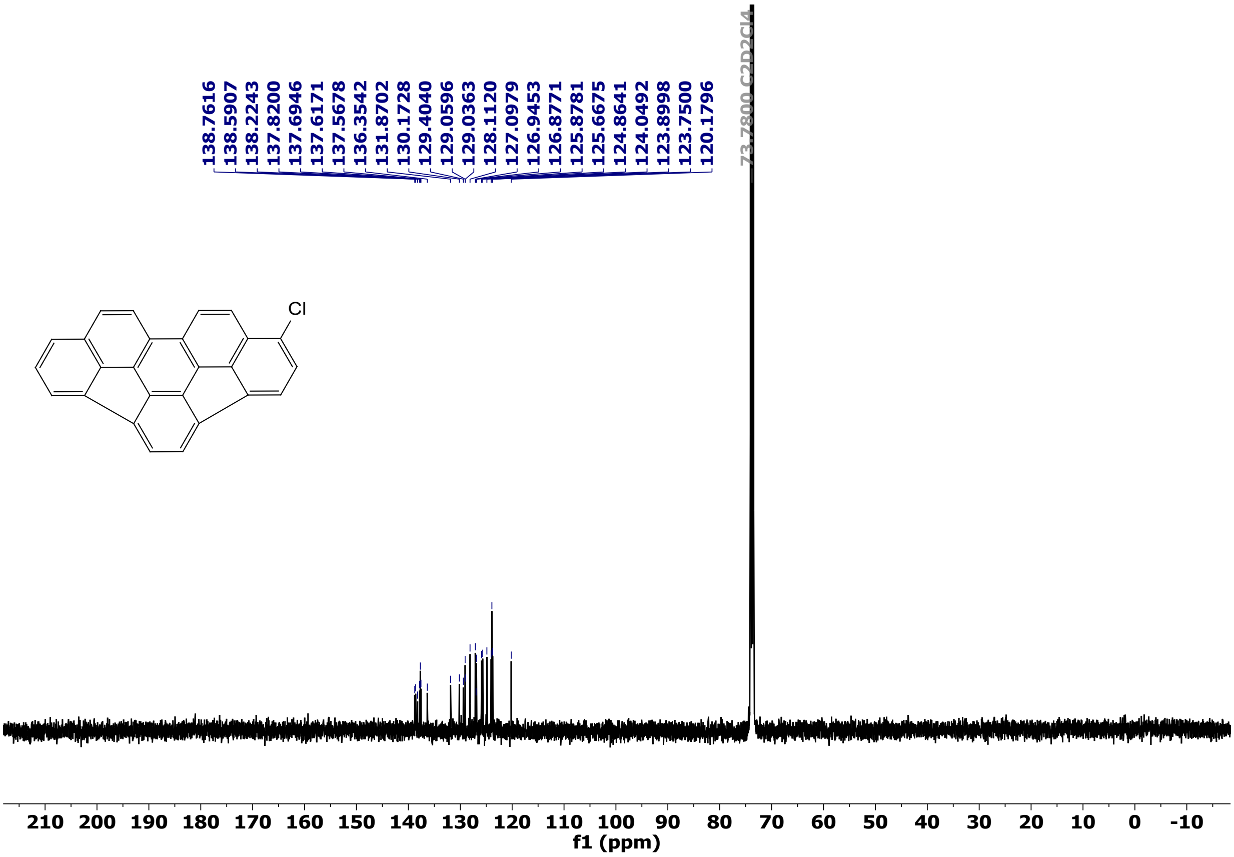

*Figure S 17. ^{13}C NMR (101 MHz, $C_2D_2Cl_4$) spectrum of compound **5b**.*

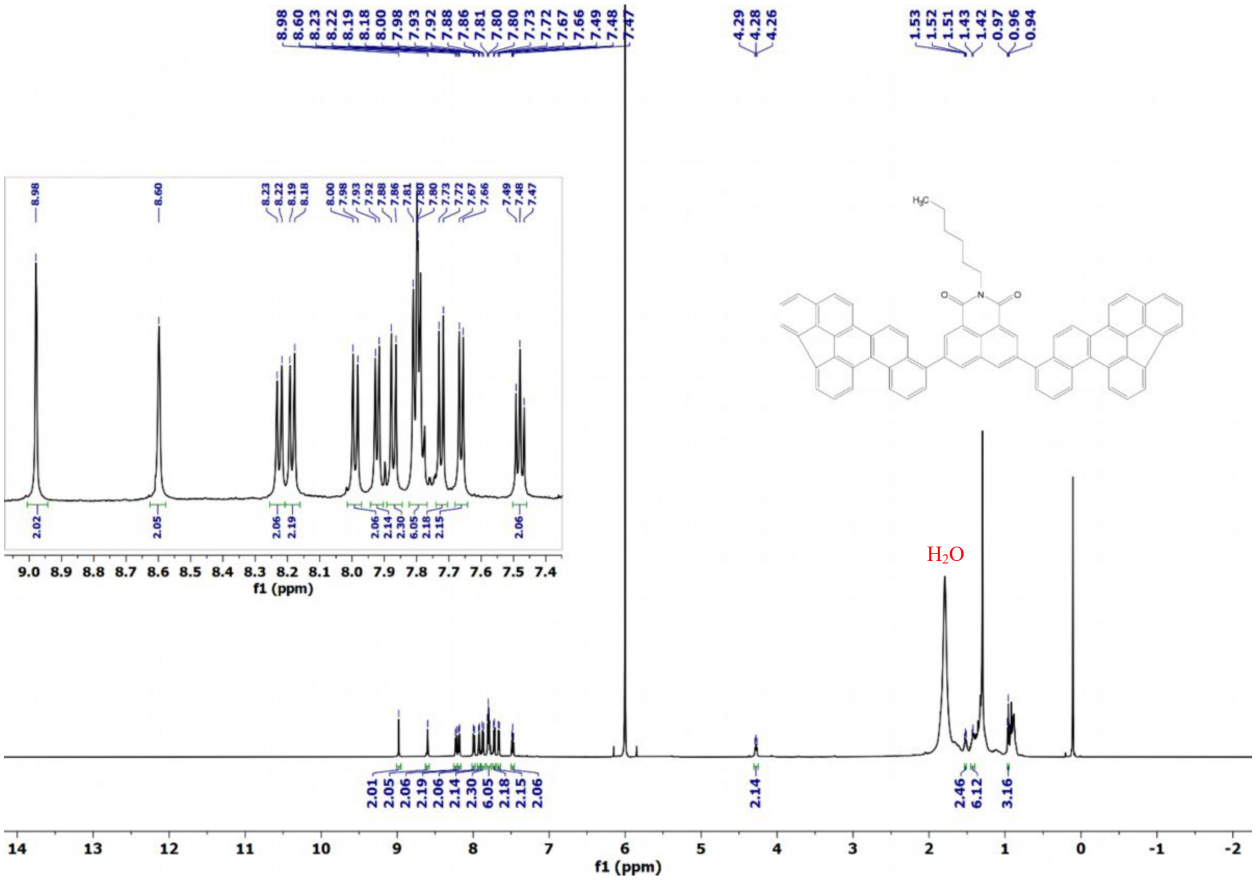

Figure S 18. ^{1}H NMR (600 MHz, $C_2D_2Cl_4$) spectrum of tweezer 7.

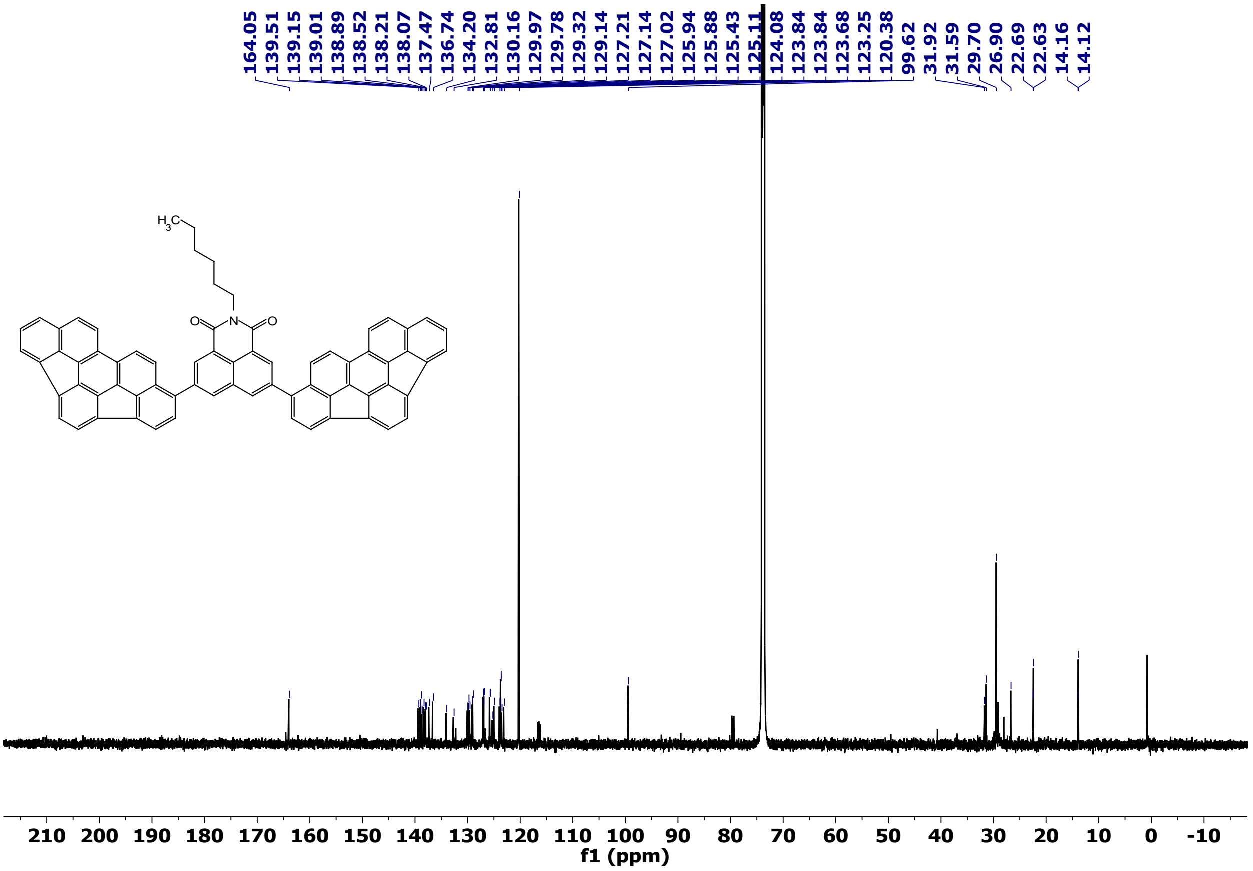

Figure S 19. ^{13}C NMR (151 MHz, $C_2D_2Cl_4$) spectrum of tweezer 7.

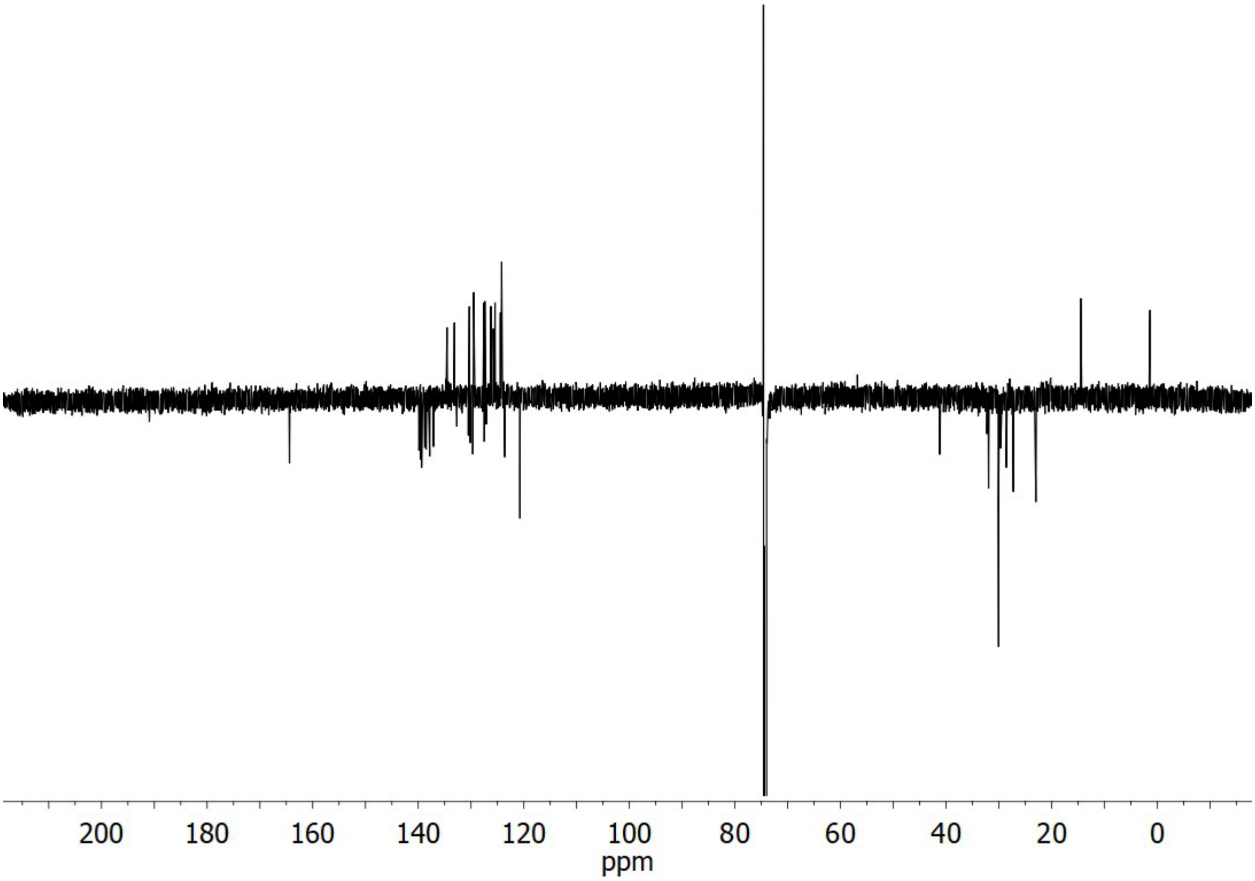

Figure S 20. DEPT-135 NMR (151 MHz, $C_2D_2Cl_4$) spectrum of tweezer 7.

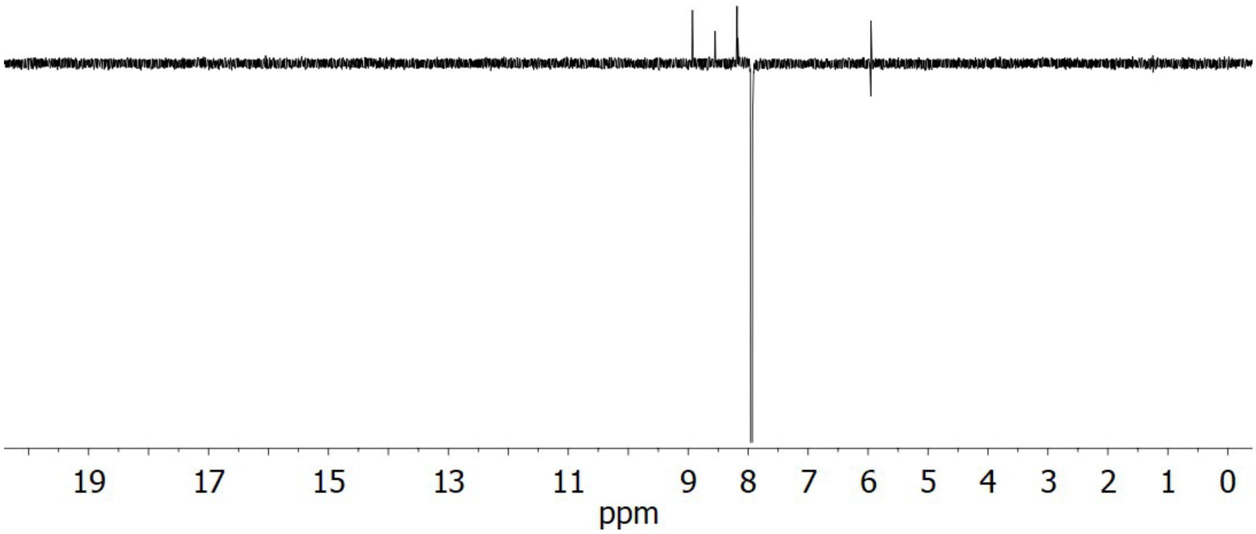

Figure S 21. ROE NMR (600 MHz, $C_2D_2Cl_4$) spectrum of tweezer 7.

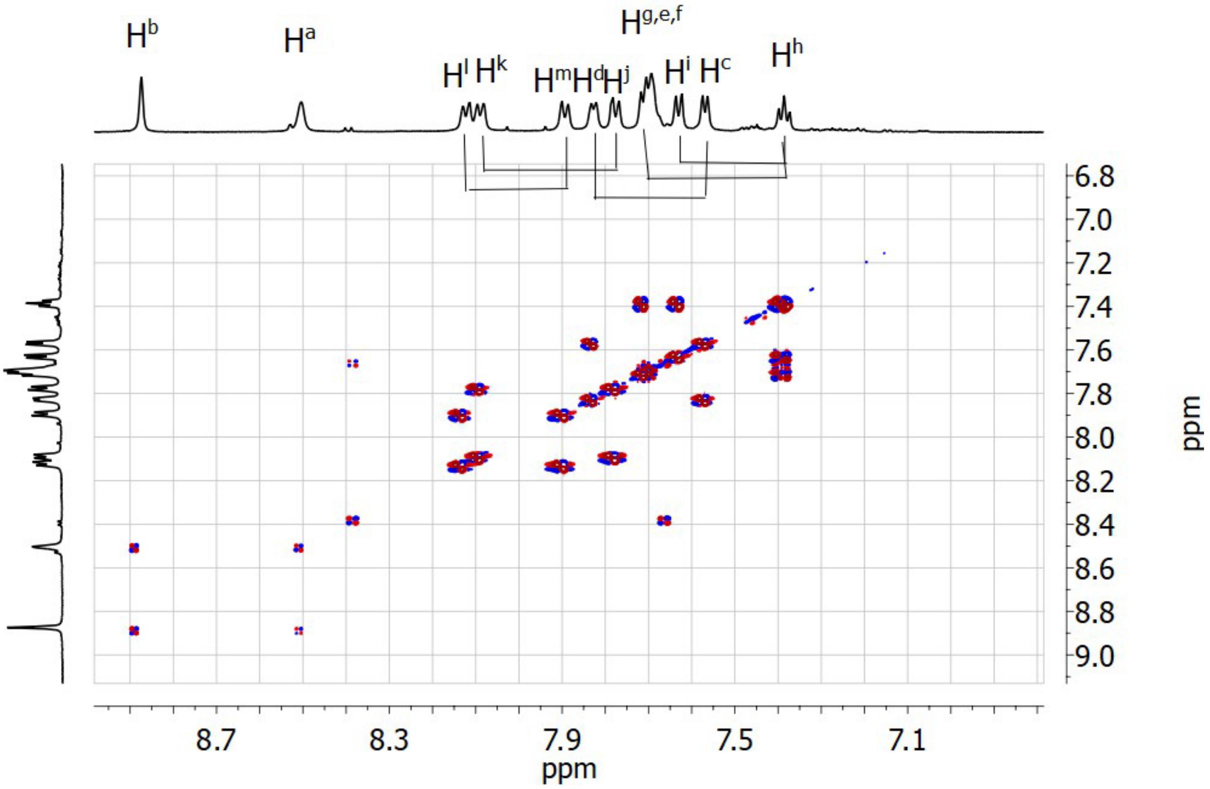

Figure S 22. ^1H-^1H COSY NMR (600 MHz, $C_2D_2Cl_4$) spectrum of tweezer 7.

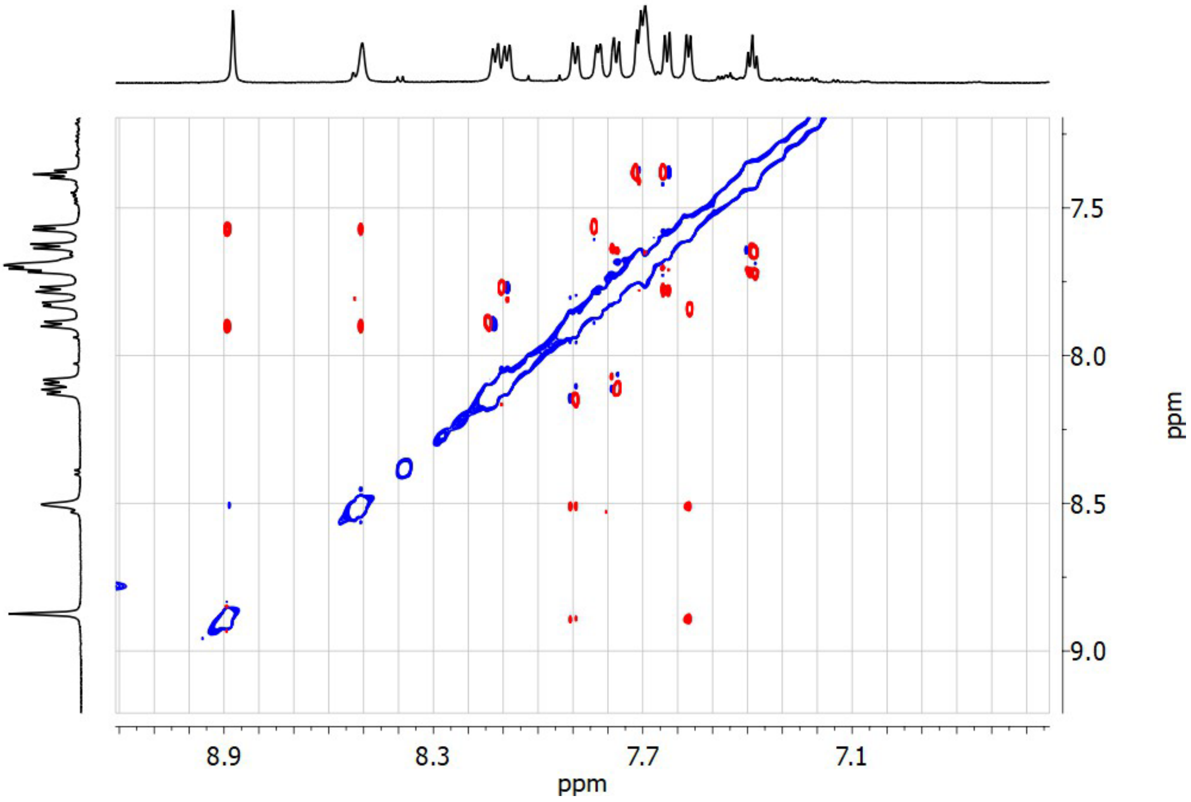

*Figure S 23. ^{1}H-^{1}H ROSY NMR (600 MHz, $C_2D_2Cl_4$) spectrum of tweezer **7**.*

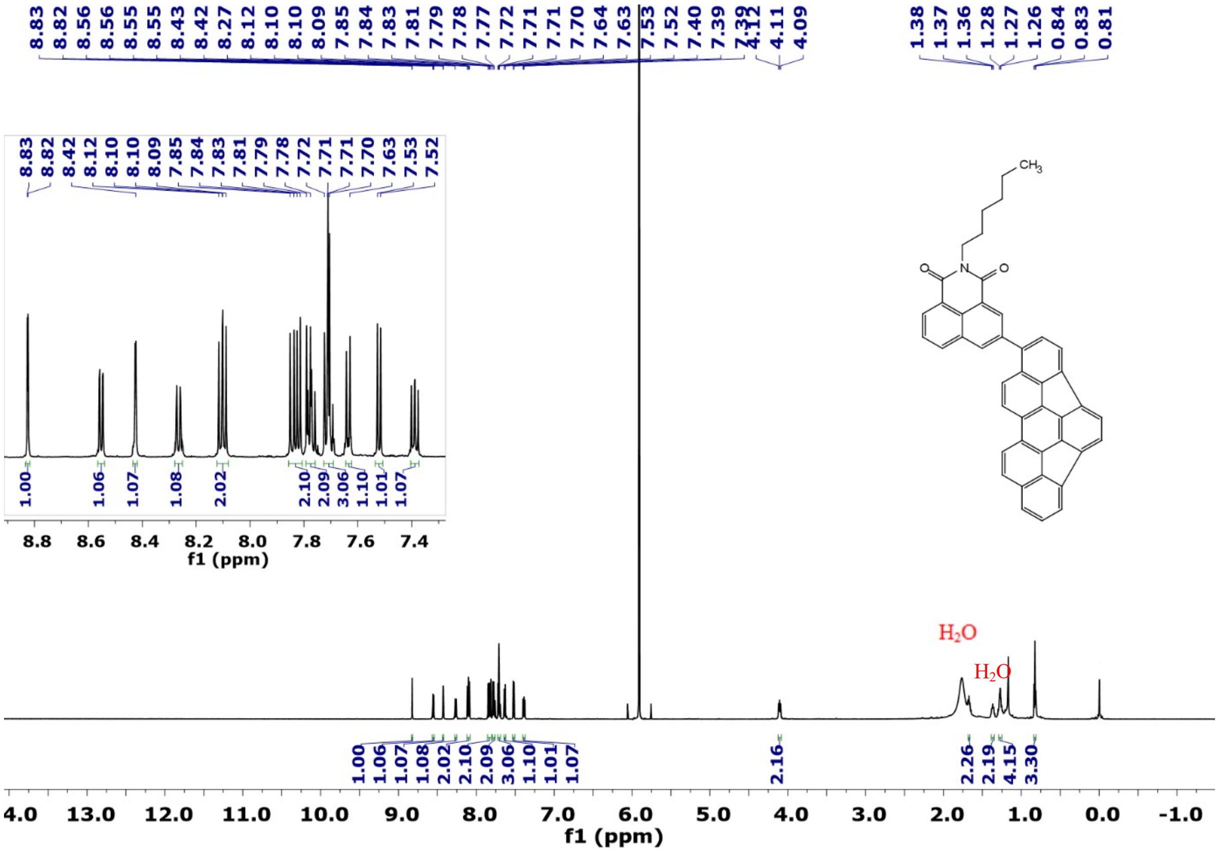

*Figure S 24. ^{1}H NMR (600 MHz, $C_2D_2Cl_4$) spectrum of **8**.*

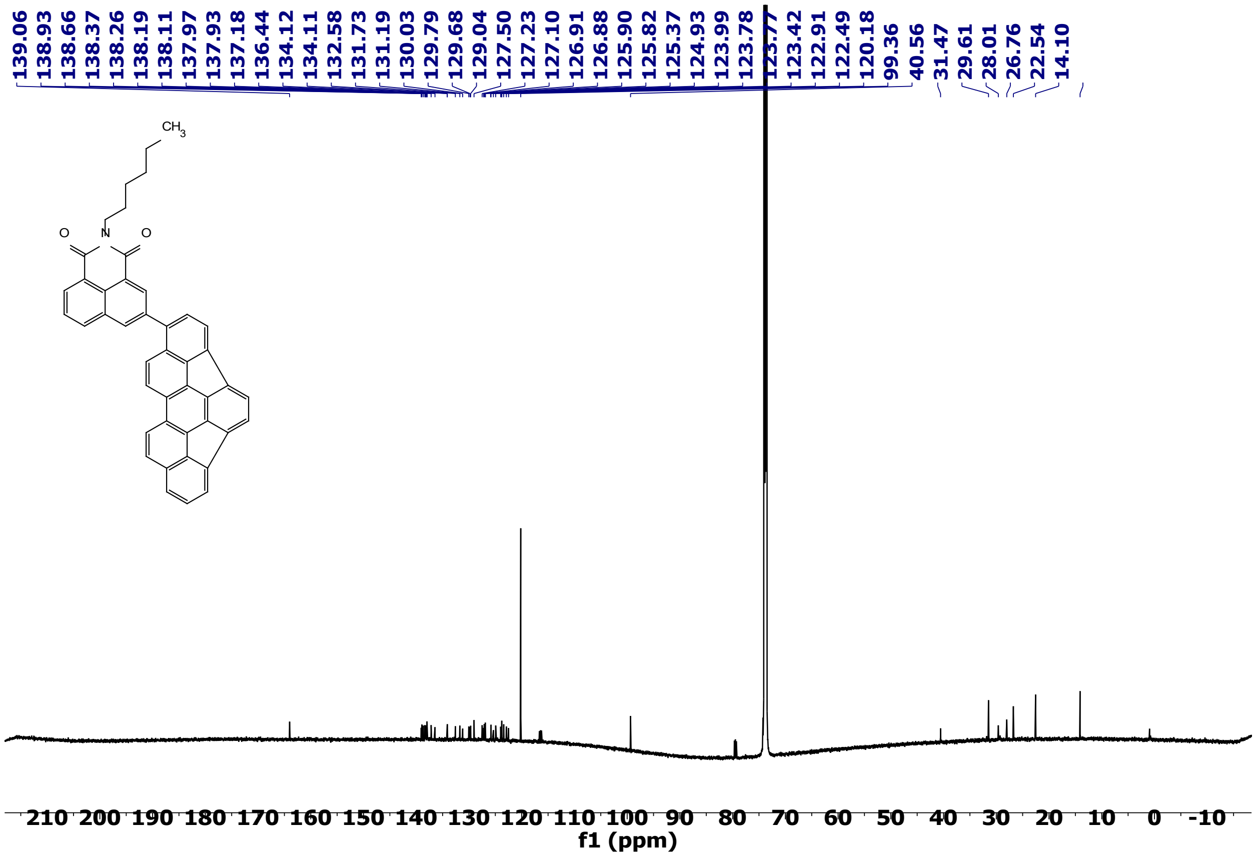

*Figure S 25. ^{13}C NMR (600 MHz, $C_2D_2Cl_4$) spectrum of **8**.*

4. UV/vis

UV/vis measurements of **8** were made at concentration 1 x 10-4 M in 1,1,2,2-tetrachloroethane (TCE) at room temperature. UV/vis measurements 200-550nm and for fluorescence measurements: 1 cm quartz cuvettes (Hellma), λ_{ex} 392nm, slit 2/2, λ_{em} 450-720 nm.

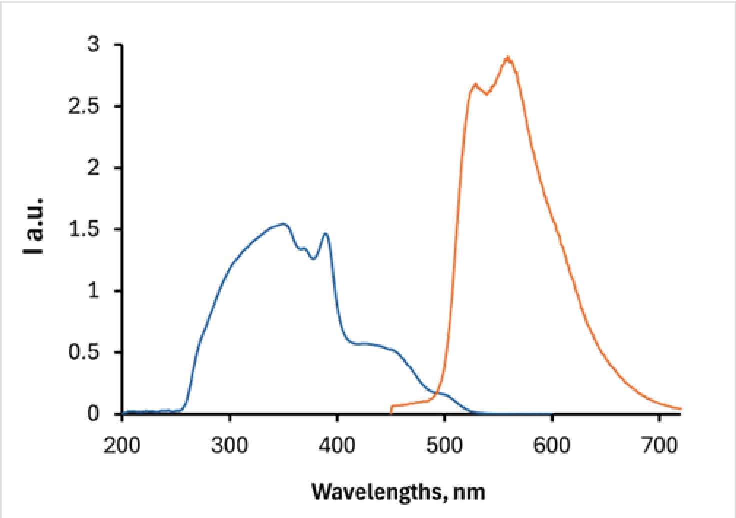

*Figure S 26. Excitation-emission (λ_{ex}392nm) of **8** in TCE.*

5. Fluorescence Titrations

Fluorescence titrations were carried out with ICP-**6** (1.0×10^{-5} M), tweezer **7** and **8** (1.0×10^{-6} M) as a host solution 1,1,2,2-tetrachloroethane (TCE) at room temperature. Measurements were made to keep host concentrations constant while adding an increasing concentration of C60 or C70 (2.0×10^{-4} M) to the cuvette. Measurements were made to keep host concentrations constant while adding an increasing concentration of C60 or C70 (2.0×10^{-4} M) to the cuvette. Measurements were made to keep host concentrations constant while adding an increasing concentration of C60 or C70 (2.0×10^{-4} M) to the cuvette. The following setup parameters were used for fluorescence titration experiments: 1 cm quartz cuvettes (Hellma), λ_{ex} 392nm, slit 2/2, λ_{em} 480-680 nm; for UV/vis measurements 250-500nm.

The resulting data were imported into the HypSpec program and fitted to obtain stability constants for the host and guest using a 1:1 binding model. Due to the inner filter effect caused by the overlap of the absorption bands of the guests (C60 and C70) with the excitation wavelength of 392 nm, the results are presented as a qualitative analysis.

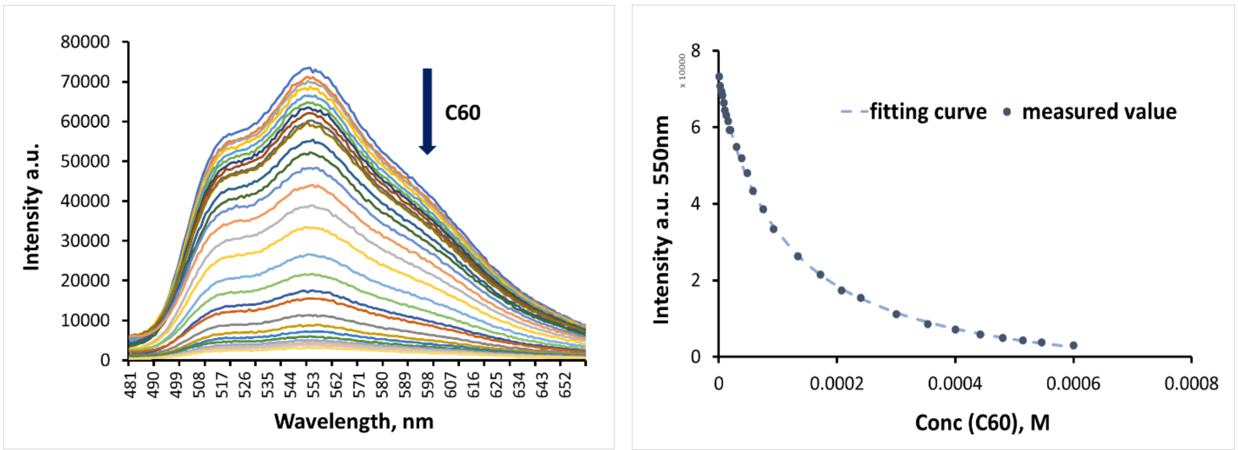

*Figure S 27. Fluorescence titration of C60 (0.20mM) into ICP **6** (0.001mM) λ_{ex}388nm in TCE and the fit curve.*

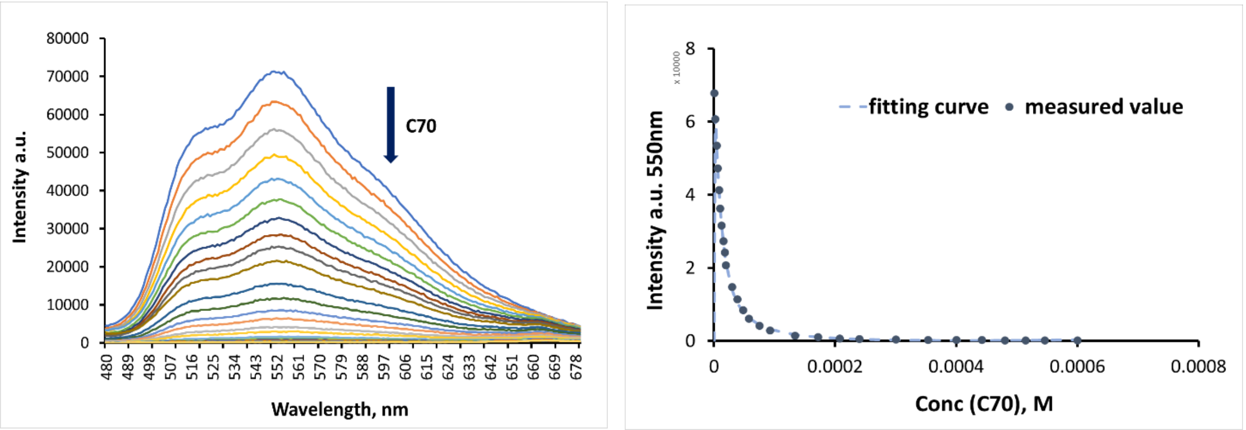

*Figure S 28. Fluorescence titration of C70 (0.20mM) into ICP **6** (0.001mM) λ_{ex}388nm in TCE and the fit curve.*

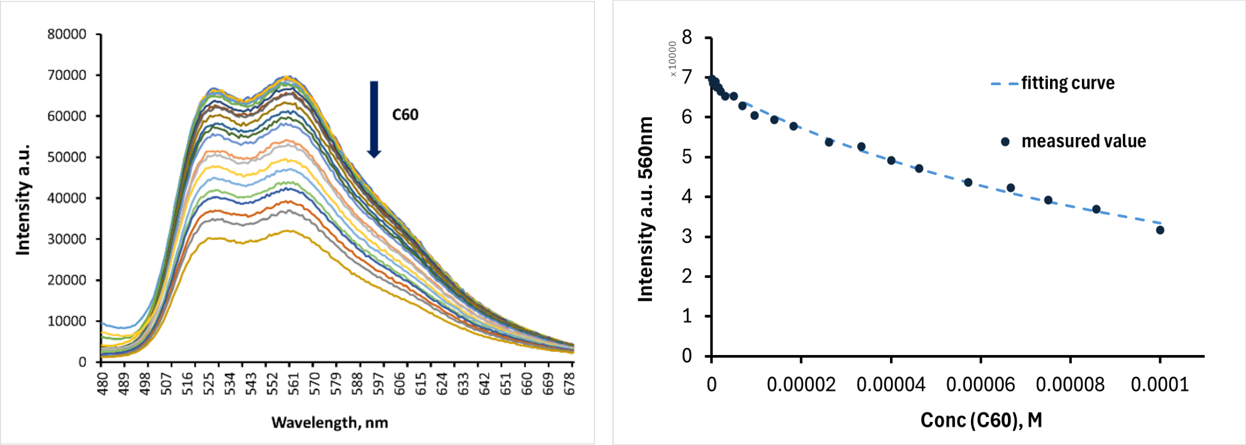

Figure S 29. Fluorescence titration of C60 (0.20mM) into tweezer 7 (0.001mM) λ_{ex}392nm in TCE and the fit curve.

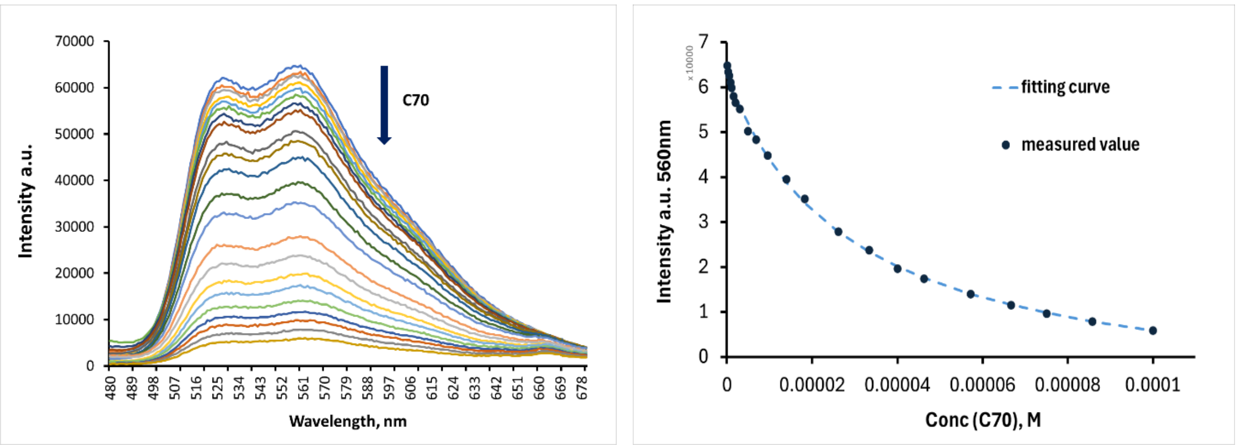

Figure S 30. Fluorescence titration of C70 (0.20mM) into tweezer 7 (0.001mM) λ_{ex}392nm in TCE and the fit curve.

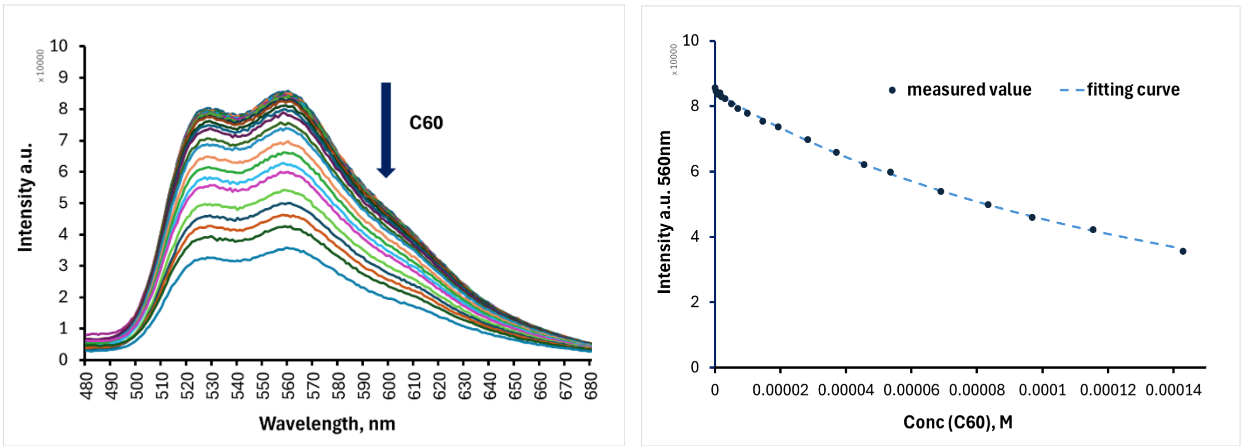

*Figure S 31. Fluorescence titration of C70 (0.20mM) into **8** (0.001mM) λ_{ex}392nm in TCE and the fit curve.*

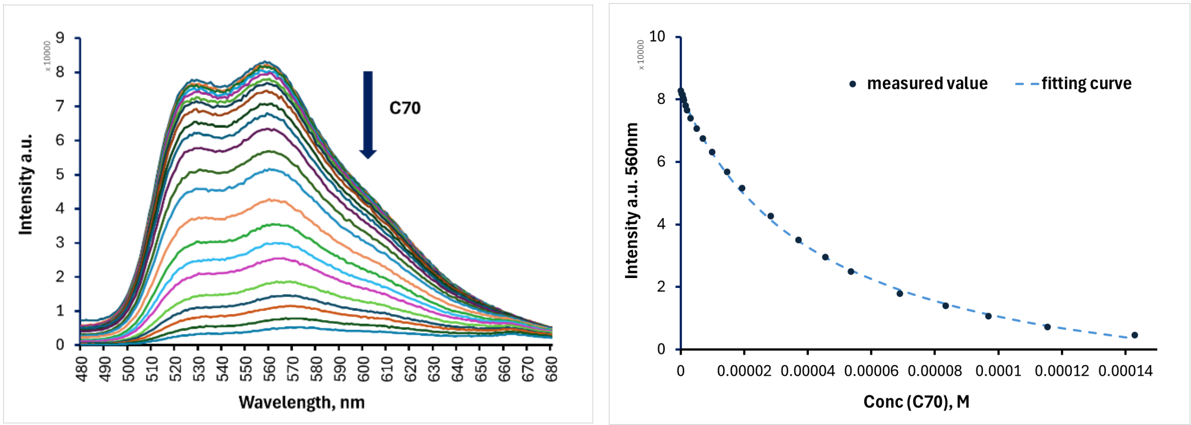

*Figure S 32. Fluorescence titration of C70 (0.20mM) into **8** (0.001mM) λ_{ex}392nm in TCE and the fit curve.*

6. NMR Titrations

NMR titrations were carried out with a 1.0×10^{-3} M ICP-**6** and tweezer **7** and **8** (4.0×10^{-4} M) as a host solution in d2-1,1,2,2-tetrachloroethane (TCE-d2). Guest solution aliquots in TCE-d2 were added to the host and spectra was recorded at 25 °C. Binding constants were calculated by a global analysis of all shifted peaks using the bind-fit software from supramolecular.org with a 1:1 model.

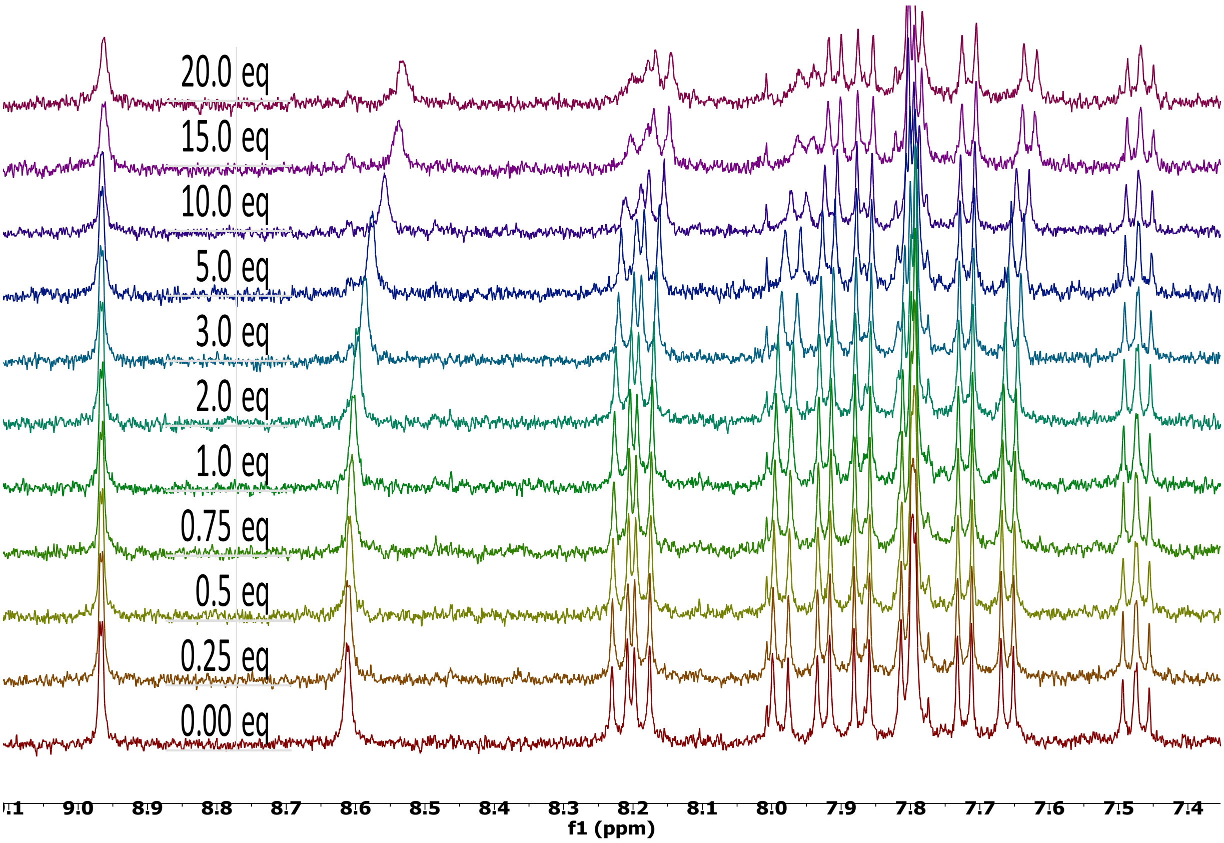

Figure S 33. ^{1}H NMR (400 MHz, $C_2D_2Cl_4$) titration spectra of the C_{60} (28mM) into tweezer 7 (0.6mM) in d2-TCE.

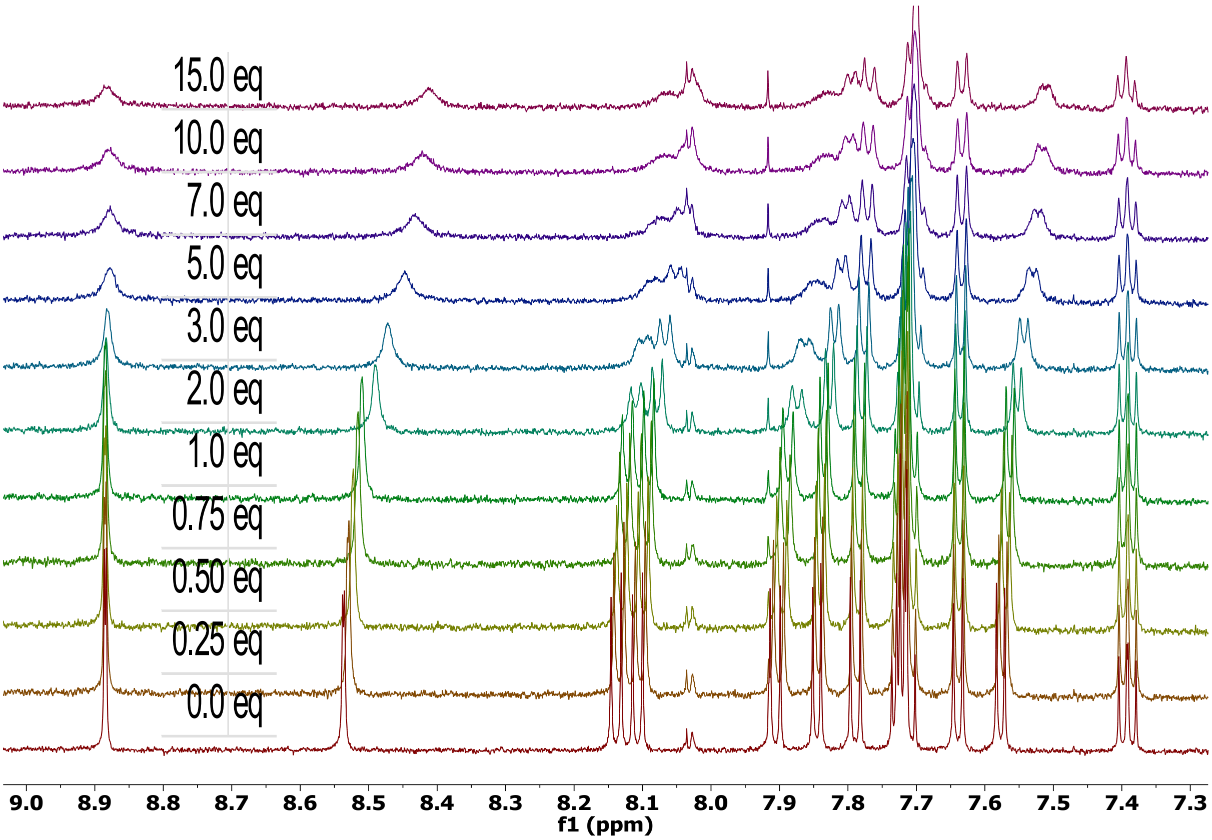

*Figure S 34. ^{1}H NMR (600 MHz, $C_2D_2Cl_4$) titration spectra of the C_{70} (8mM) into tweezer **7** (0.4mM) in d2-TCE.*

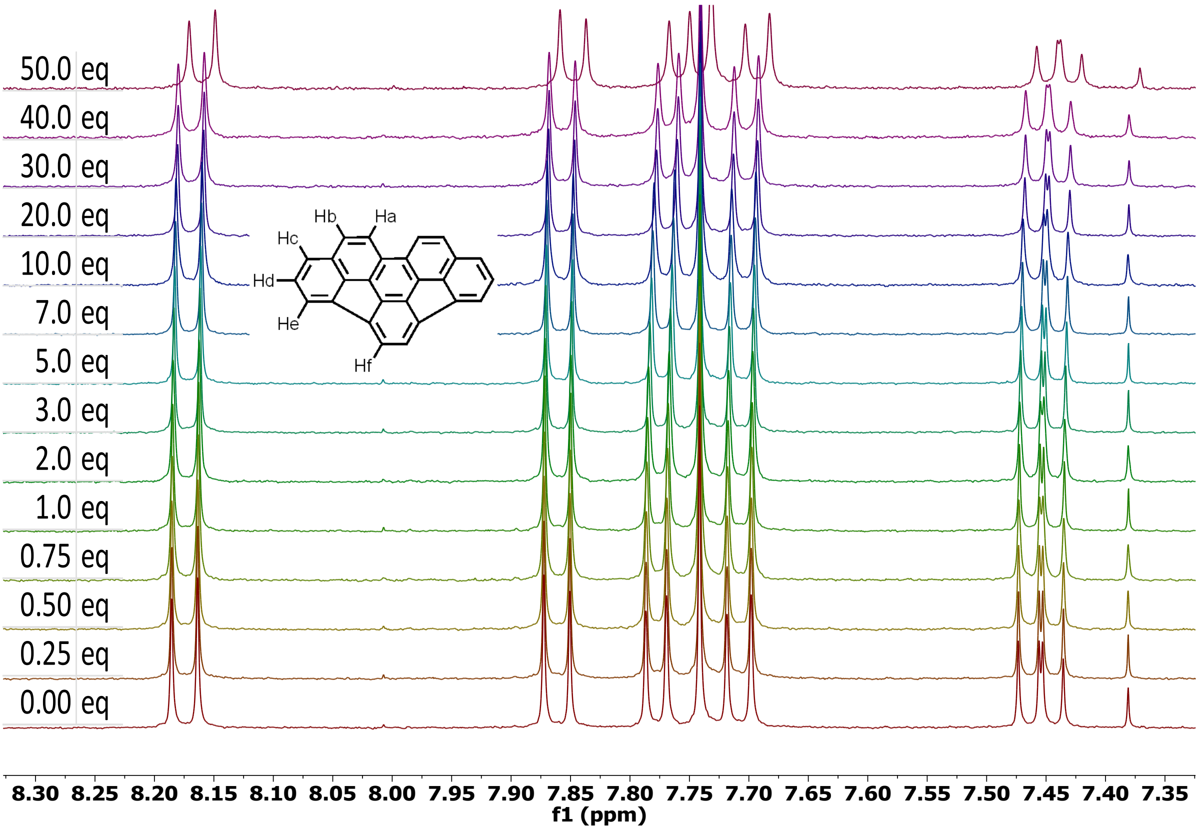

*Figure S 35. ^{1}H NMR (400 MHz, $C_2D_2Cl_4$) titration spectra of the C_{60} (50mM) into ICP **6** (1mM).*

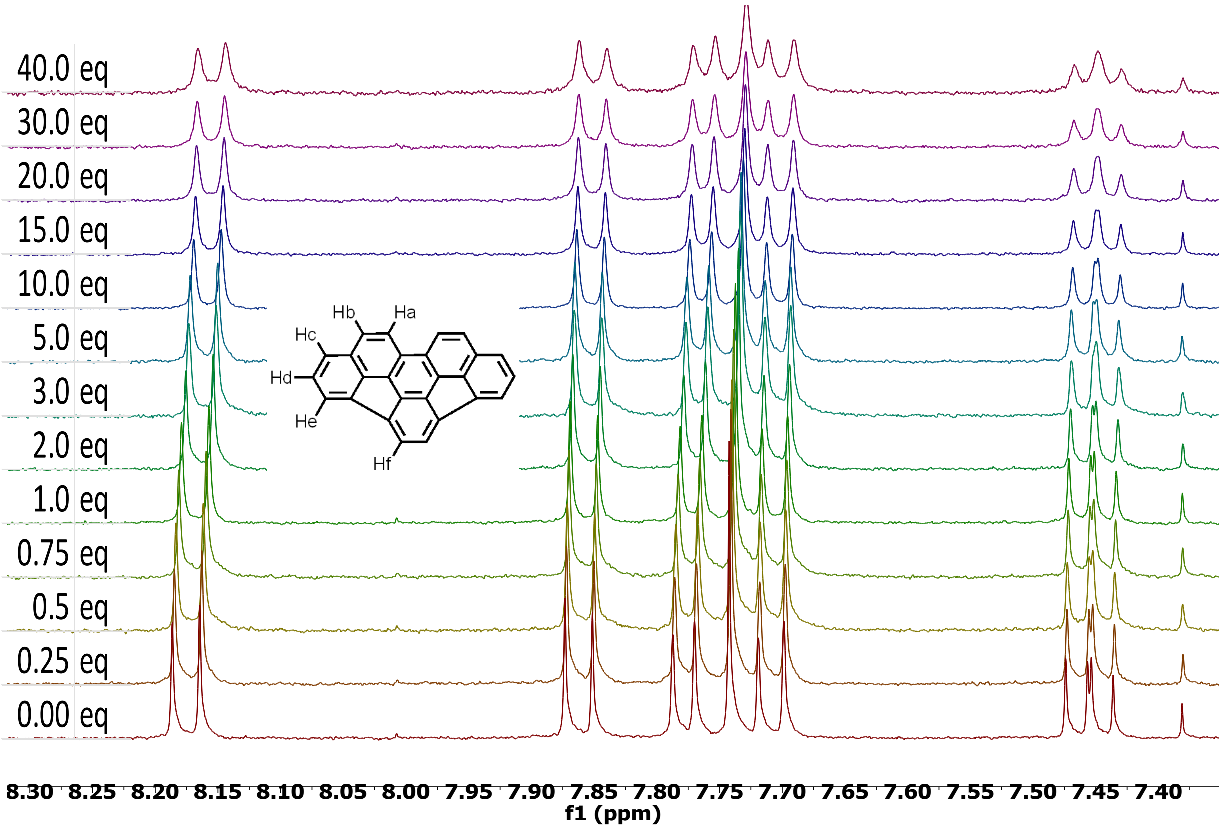

*Figure S 36. ^{1}H NMR (400 MHz, $C_2D_2Cl_4$) titration spectra of the C_{70} (50mM) into ICP **6** (1mM) in d2-TCE.*

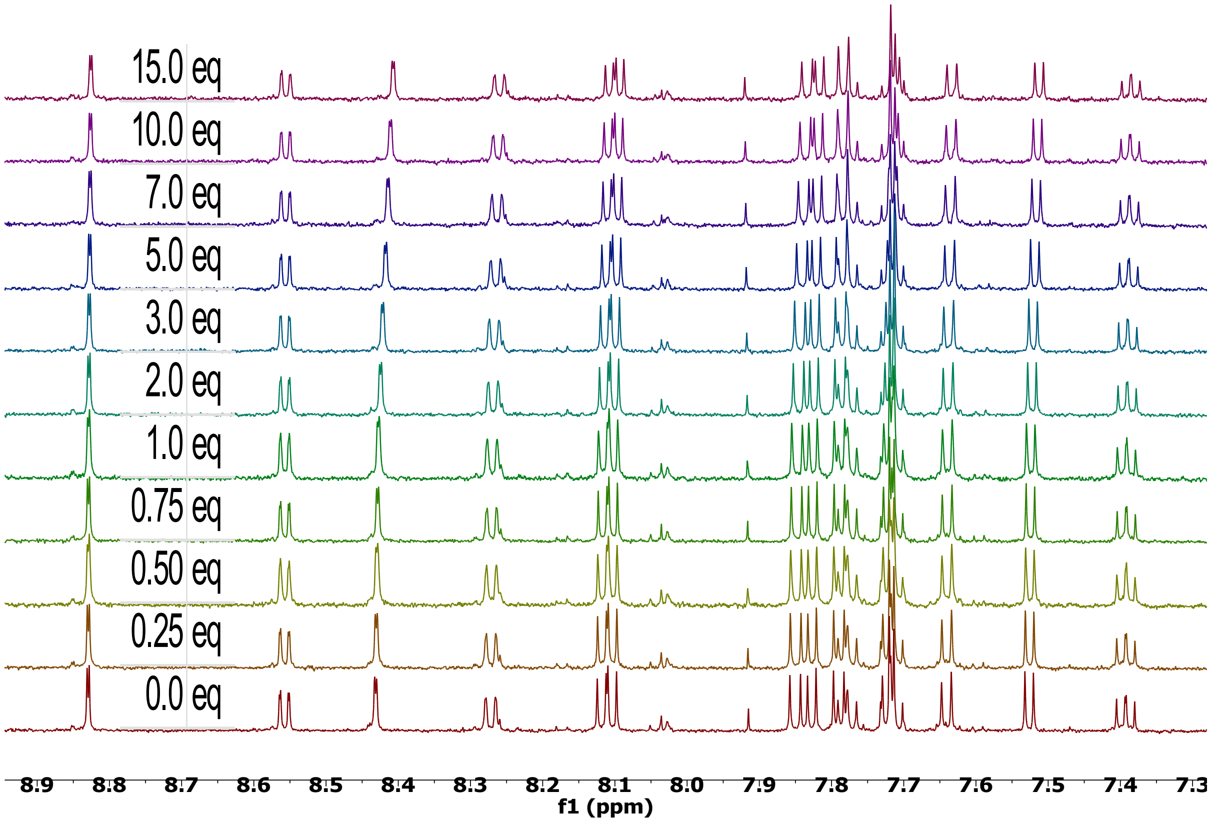

*Figure S 37. ^{1}H NMR (600 MHz, $C_2D_2Cl_4$) titration spectra of the C60 (8mM) into **8** (0.4mM).*

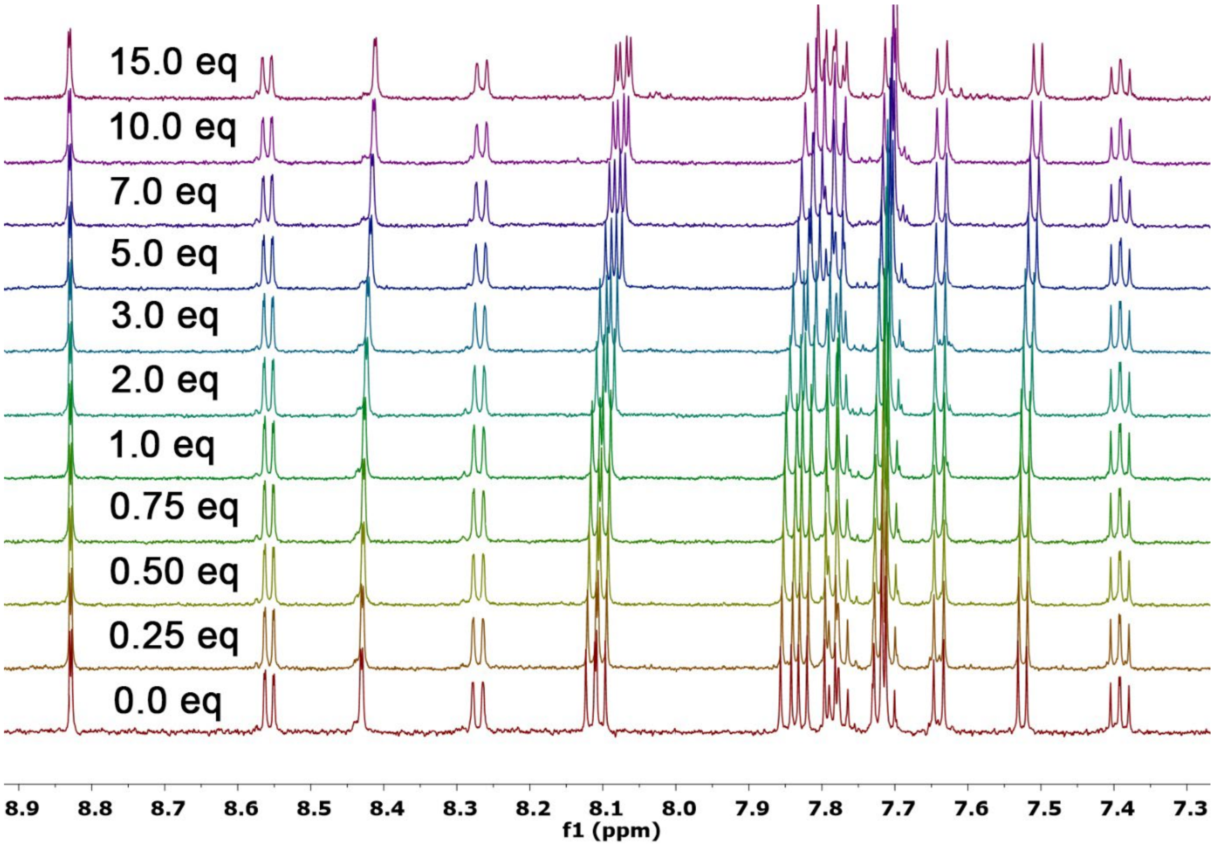

*Figure S 38. ^{1}H NMR (600 MHz, $C_2D_2Cl_4$) titration spectra of the C70 (8mM) into **8** (0.4mM).*

Binding constant of ICP **6** for C70 (670 M^{-1}) http://app.supramolecular.org/bindfit/view/dd0a8f2f-afac-4655-89cc-646647047f0a

For ICP **6** C60 (140 M^{-1}) http://app.supramolecular.org/bindfit/view/ccab9bda-432e-4f5e-91cb-de660cbc67d6

Tweezer **7** C60 (43 M^{-1}) http://app.supramolecular.org/bindfit/view/83dc4dca-107b-4bc5-a55c-879a9f4a9be0

for Tweezer **7** C70 (2147 M^{-1}) http://app.supramolecular.org/bindfit/view/bf2da8d1-be71-46e6-9666-a444f9c2625b

8 for C60 (314.88 M^{-1}) http://app.supramolecular.org/bindfit/view/9db896fb-94d3-4e37-8d71-185118c577ed

8 for C70 (555.08 M^{-1}) http://app.supramolecular.org/bindfit/view/50fcf47d-e770-4c22-963d-e92fb147d90d

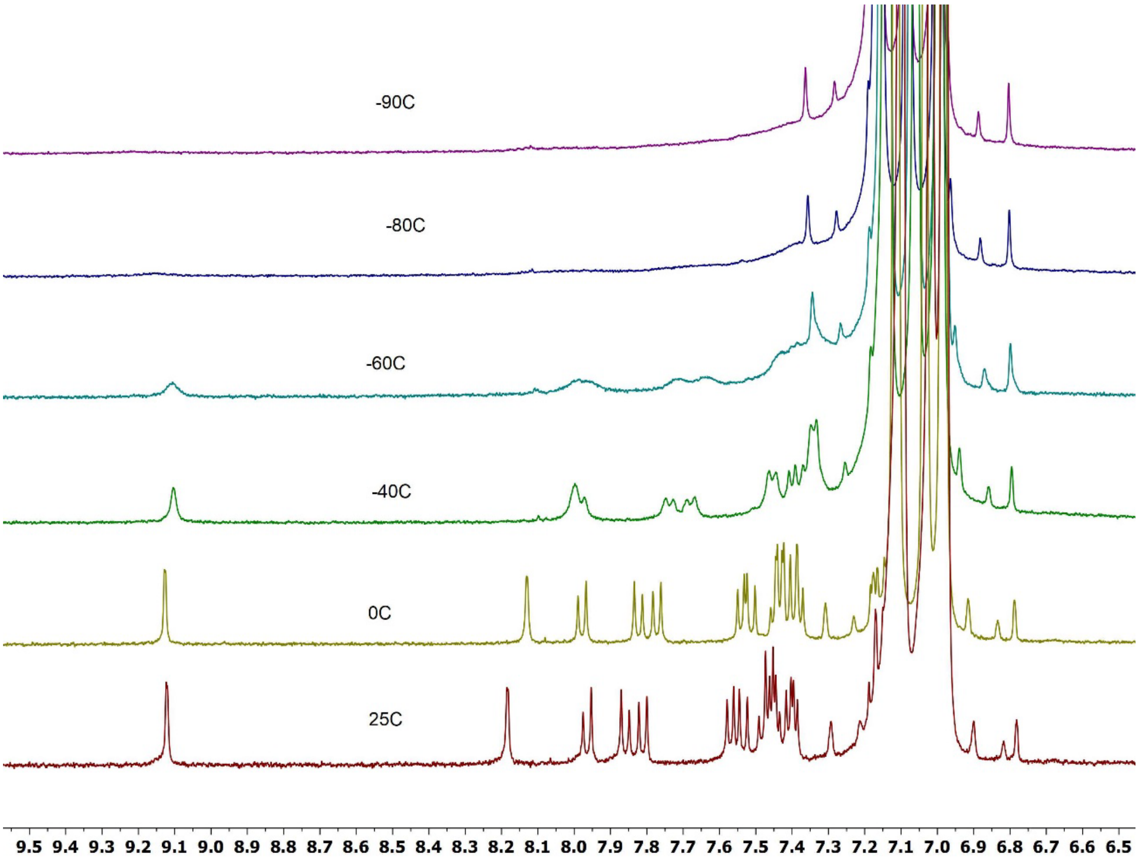

Figure S 39. ^{1}H NMR (400 MHz, Toluene-d8) temperature dependent spectra of the tweezer 7.

7. DFT Calculations

Table S1. Calculated energies of three different conformations of complex 7•C_{70}.

Conformation	Absolute energy (Hartree)	Relative energy (kcal/mol)
1	-5366.15605071660	0.14
2	-5366.15625858227	0.00
3	-5366.15247322445	2.38

Table S2. Calculated buried surface area in three different conformations of the complex 7•C_{70}.

	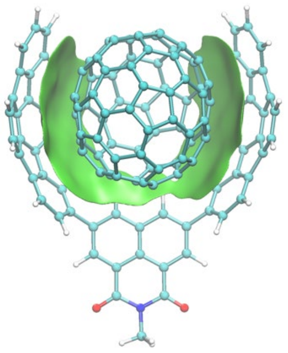	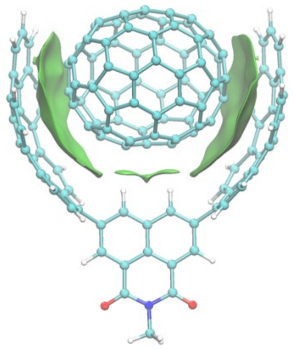	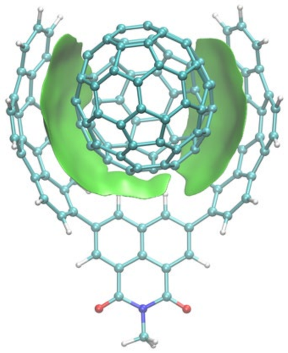
Binding mode	1	2 (most favorable))	3
Surf.Area	3647.905	3676.846	3702.9732
Buried	812.1652	783.2242	757.097

Optimized structure of tweezer **7** with C_{60}.

C60_7	-4985.069305
C60	-2285.872254
7	-2699.143038

C	4.666634000	1.145018000	0.805386000
C	3.854398000	0.998047000	-0.382132000
C	3.681654000	2.075803000	-1.244631000
C	4.310376000	3.342879000	-0.949150000
C	5.090330000	3.484521000	0.193160000
C	5.274950000	2.364747000	1.086529000
C	2.383102000	2.349304000	-1.820130000
C	3.410962000	4.400963000	-1.347907000
C	5.004406000	4.690354000	0.984568000
C	5.289181000	2.876638000	2.439784000
C	4.131856000	5.705449000	0.606070000
C	3.320955000	5.558782000	-0.582636000
C	3.338171000	6.384636000	1.607261000
C	2.026551000	6.146045000	-0.316514000
C	2.215590000	3.786256000	-1.882582000
C	2.036455000	6.655118000	1.036759000
C	0.972433000	4.352521000	-1.625395000
C	1.302224000	1.534873000	-1.502518000
C	5.121673000	4.313465000	2.376967000
C	4.688704000	2.149069000	3.461240000
C	3.894294000	2.829008000	4.461646000
C	0.876092000	5.554632000	-0.826890000

C	4.358374000	4.966795000	3.338852000
C	0.007882000	2.123826000	-1.237987000
C	-0.153715000	3.504765000	-1.298348000
C	-0.947394000	4.184873000	-0.296370000
C	3.449403000	6.022719000	2.945958000
C	-1.547642000	3.456857000	0.725732000
C	3.732598000	4.209635000	4.401547000
C	2.437487000	4.798284000	4.666671000
C	-0.310089000	5.450641000	-0.006031000
C	-0.300763000	5.942673000	1.294395000
C	1.354960000	3.983690000	4.983195000
C	0.053530000	4.256135000	4.412220000
C	-1.537512000	3.967715000	2.079346000
C	2.262447000	5.918637000	3.767891000
C	0.895981000	6.557239000	1.826134000
C	-0.925600000	5.185972000	2.357854000
C	1.011543000	6.179867000	3.218414000
C	-0.114811000	5.332708000	3.547443000
C	4.042423000	0.387858000	1.869258000
C	4.053368000	0.880637000	3.170753000
C	1.478870000	0.415970000	-0.602593000
C	2.728863000	0.151465000	-0.054143000
C	2.768156000	1.982025000	4.789815000
C	1.523561000	2.547814000	5.045809000
C	-0.582458000	2.989260000	4.122513000
C	-1.361567000	2.847873000	2.978825000
C	-1.379867000	2.020407000	0.788296000
C	-0.615961000	1.367271000	-0.173168000
C	2.866491000	0.778258000	3.992590000
C	0.325873000	1.933161000	4.514006000
C	-1.265484000	1.644005000	2.180880000
C	0.293820000	0.314151000	0.219120000
C	2.844625000	-0.223548000	1.337598000
C	0.403804000	-0.049288000	1.556462000
C	-0.391056000	0.629127000	2.557035000
C	0.419659000	0.776808000	3.746894000
C	1.715112000	0.188499000	3.480531000
C	1.704446000	-0.321481000	2.127193000
C	-2.199024000	-0.961685000	-2.324121000
C	-2.562612000	-1.592067000	-1.145519000
C	-1.687325000	-2.434210000	-0.405297000
C	-0.436380000	-2.708844000	-0.916464000
C	-0.126492000	-2.113960000	-2.180979000
C	-0.904294000	-1.183504000	-2.864217000
C	1.223787000	-2.309294000	-2.504806000
C	1.945044000	-1.507343000	-3.389410000
C	1.127497000	-0.607008000	-4.150439000
C	-0.231234000	-0.475911000	-3.917601000

C	1.766149000	-3.076257000	-1.452601000
C	3.109376000	-3.073394000	-1.281658000
C	3.892506000	-2.296116000	-2.161169000
C	3.407388000	-1.501777000	-3.200328000
C	5.113848000	-2.080605000	-1.500764000
C	6.018278000	-1.123889000	-1.954942000
C	5.642812000	-0.467393000	-3.179140000
C	4.392672000	-0.618622000	-3.752135000
C	0.849726000	-3.289942000	-0.403845000
C	1.435327000	-3.608770000	0.826032000
C	2.858232000	-3.609669000	1.006627000
C	3.732752000	-3.288324000	-0.037213000
C	5.096242000	-2.688091000	-0.204398000
C	6.176770000	-2.412859000	0.606869000
C	7.175311000	-1.522524000	0.141707000
C	7.109341000	-0.836974000	-1.071545000
C	7.526138000	6.401291000	-1.461827000
C	7.681751000	7.194295000	-0.324011000
C	6.775777000	8.209548000	0.068959000
C	5.699885000	8.496593000	-0.744274000
C	4.397550000	9.229333000	-0.618327000
C	3.574046000	9.738885000	0.392418000
C	2.155980000	9.869336000	0.215761000
C	1.523923000	9.498197000	-0.976342000
C	4.744549000	6.193786000	-4.085472000
C	5.984093000	5.975818000	-3.509458000
C	6.441732000	6.699304000	-2.351731000
C	5.635662000	7.770829000	-1.977271000
C	3.854014000	7.209747000	-3.606993000
C	4.428437000	8.039068000	-2.644071000
C	3.737505000	8.963133000	-1.833007000
C	2.400251000	9.089823000	-2.000276000
C	0.116877000	6.508838000	-4.223332000
C	1.480169000	6.470049000	-4.463945000
C	2.395552000	7.342604000	-3.786715000
C	1.770236000	8.293069000	-2.978486000
C	-0.467454000	7.375293000	-3.238067000
C	0.410738000	8.273342000	-2.639264000
C	0.179493000	9.004277000	-1.428912000
C	-1.085077000	8.901212000	-0.889860000
C	-2.051822000	8.094855000	-1.553214000
C	-1.771736000	7.335339000	-2.676500000
C	9.689834000	5.076849000	-1.266915000
C	8.306943000	5.150598000	-1.559452000
C	7.612820000	3.974144000	-1.778104000
C	8.216569000	2.716636000	-1.608159000
C	9.593393000	2.652645000	-1.275765000
C	10.324954000	3.853313000	-1.148935000

C	7.455104000	1.533590000	-1.617127000
C	8.013276000	0.311723000	-1.283775000
C	9.399839000	0.250538000	-1.006459000
C	10.176063000	1.394851000	-1.014390000
C	11.616469000	1.312862000	-0.699374000
N	12.322011000	2.523838000	-0.658100000
C	11.769336000	3.803904000	-0.833713000
O	12.453041000	4.809985000	-0.729652000
O	12.193129000	0.258602000	-0.481593000
H	-2.005815000	-2.806451000	0.564916000
H	-3.547357000	-1.392824000	-0.732400000
H	0.825623000	-3.746152000	1.715648000
H	3.226080000	-3.745031000	2.020744000
H	6.250990000	-2.801616000	1.619071000
H	7.982790000	-1.268076000	0.823346000
H	6.340925000	0.221048000	-3.645594000
H	6.386997000	1.607842000	-1.796224000
H	6.549626000	4.010039000	-1.976957000
H	10.275120000	5.980773000	-1.123382000
H	9.874657000	-0.697920000	-0.771260000
H	8.480430000	6.936346000	0.366592000
H	6.910608000	8.686511000	1.036122000
H	3.966987000	9.936731000	1.386655000
H	1.575613000	10.154615000	1.089647000
H	-1.352308000	9.383930000	0.046628000
H	-3.045732000	8.032959000	-1.119071000
H	6.608865000	5.190036000	-3.923128000
H	4.437912000	5.544240000	-4.901538000
H	1.860928000	5.714527000	-5.146551000
H	-0.522332000	5.795060000	-4.737536000
H	-2.534627000	6.681062000	-3.091081000
H	-0.790483000	0.252716000	-4.499715000
H	-2.887470000	-0.268705000	-2.801428000
H	1.586785000	0.038545000	-4.894722000
H	4.151778000	-0.016595000	-4.624629000
C	13.755293000	2.417425000	-0.357012000
H	13.896937000	2.003603000	0.645711000
H	14.228874000	1.741608000	-1.073301000
H	14.188120000	3.414434000	-0.419221000

Optimized structure of tweezer **7** with C_{70}.

C70	-2666.953392
7	-2699.143038

Optimized structure of **7** with C_{70}, first conformation.

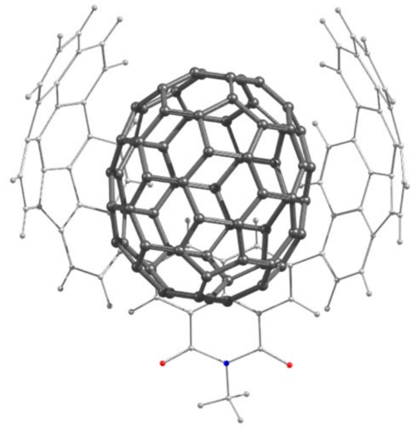

E=-5366.1560507166

C	-3.276489000	-1.262306000	-0.706397000
C	-3.178689000	-1.908788000	0.514173000
C	-2.072904000	-2.724958000	0.885390000
C	-1.076126000	-2.956546000	-0.038450000
C	-1.249676000	-2.337772000	-1.318741000
C	-2.245810000	-1.431484000	-1.668561000
C	-0.097650000	-2.487639000	-2.102241000
C	0.249687000	-1.652277000	-3.164112000
C	-0.803230000	-0.763677000	-3.565044000
C	-1.999721000	-0.685099000	-2.871649000
C	0.792245000	-3.264919000	-1.333411000
C	2.105381000	-3.246925000	-1.658263000
C	2.513829000	-2.433357000	-2.735990000
C	1.684431000	-1.620815000	-3.508766000
C	3.892756000	-2.234463000	-2.564587000
C	4.574844000	-1.262247000	-3.291990000
C	3.777575000	-0.557459000	-4.259616000
C	2.403082000	-0.703916000	-4.343817000
C	0.315087000	-3.524235000	-0.034102000
C	1.308190000	-3.871424000	0.890078000
C	2.700450000	-3.861702000	0.543281000
C	3.138373000	-3.498358000	-0.736267000
C	4.350291000	-2.891031000	-1.376691000
C	5.660579000	-2.651799000	-1.021973000
C	6.427694000	-1.753379000	-1.803483000
C	5.924827000	-1.021855000	-2.880330000
C	6.717322000	6.166408000	-2.678469000
C	7.367022000	6.684237000	-1.556807000
C	6.820819000	7.672372000	-0.701378000
C	5.602431000	8.229873000	-1.023036000
C	4.565022000	9.070047000	-0.339128000
C	4.211846000	9.459368000	0.958749000
C	2.860527000	9.797401000	1.304093000
C	1.826811000	9.764774000	0.359971000

C	3.221172000	6.780461000	-4.122488000
C	4.520006000	6.302626000	-4.056562000
C	5.455600000	6.731682000	-3.051893000
C	5.013539000	7.781465000	-2.250104000
C	2.735598000	7.789287000	-3.227342000
C	3.727972000	8.321438000	-2.404458000
C	3.515591000	9.137305000	-1.273883000
C	2.244451000	9.465963000	-0.950806000
C	-1.074913000	7.752992000	-2.640885000
C	0.113308000	7.578421000	-3.331952000
C	1.347337000	8.154226000	-2.880094000
C	1.201668000	8.983387000	-1.767530000
C	-1.145662000	8.464752000	-1.395556000
C	0.037173000	9.078864000	-0.994926000
C	0.337606000	9.566599000	0.318975000
C	-0.700092000	9.542076000	1.226157000
C	-1.967135000	9.045971000	0.807788000
C	-2.200550000	8.504866000	-0.445007000
C	8.638182000	4.770335000	-3.542029000
C	7.258988000	4.923207000	-3.266026000
C	6.431412000	3.818353000	-3.366889000
C	6.954964000	2.527652000	-3.580218000
C	8.349885000	2.378922000	-3.798334000
C	9.168063000	3.527831000	-3.831271000
C	6.164653000	1.374831000	-3.401503000
C	6.729408000	0.110822000	-3.382319000
C	8.107485000	-0.026892000	-3.670013000
C	8.899345000	1.082984000	-3.893368000
C	10.342756000	0.908406000	-4.166550000
N	11.098547000	2.086232000	-4.281221000
C	10.612978000	3.388830000	-4.103305000
O	11.372509000	4.342364000	-4.175949000
O	10.858306000	-0.191667000	-4.287491000
C	1.150752000	-0.513665000	1.922682000
C	2.956618000	1.410759000	-1.302572000
C	3.595011000	5.399053000	-0.242628000
C	2.180539000	5.938976000	3.643119000
C	0.669848000	2.283284000	4.982154000
C	0.573481000	0.967784000	4.348096000
C	1.717903000	-0.406526000	0.578885000
C	3.399294000	2.790894000	-1.491430000
C	3.303779000	6.146166000	0.978867000
C	1.554263000	5.018929000	4.594534000
C	-1.922402000	2.836445000	0.879722000
C	5.195990000	0.938175000	3.733012000
C	-1.361901000	2.940347000	-0.449792000
C	5.756586000	1.041736000	2.404803000
C	-0.920872000	4.303814000	-0.643398000

C	6.199108000	2.405723000	2.212793000
C	-1.207844000	5.041311000	0.566330000
C	5.910670000	3.144617000	3.421065000
C	-1.827294000	4.134996000	1.506762000
C	5.291269000	2.237385000	4.360478000
C	-1.704194000	1.686263000	1.633681000
C	4.072122000	0.144841000	3.949412000
C	-0.607598000	1.890126000	-0.964629000
C	5.169496000	0.346737000	1.350740000
C	0.254708000	4.557964000	-1.344330000
C	6.033663000	3.017983000	0.973983000
C	-0.306380000	5.997518000	1.021800000
C	5.470154000	4.463758000	3.338422000
C	-1.521187000	4.228861000	2.862594000
C	4.257714000	2.688030000	5.177099000
C	-0.915076000	0.606321000	1.096368000
C	3.462941000	-0.566703000	2.852149000
C	0.611860000	2.157622000	-1.685779000
C	4.991291000	0.989025000	0.072069000
C	1.187036000	5.546752000	-0.862839000
C	5.569469000	4.382312000	0.893416000
C	0.012214000	6.088451000	2.424055000
C	4.393088000	4.922446000	4.180961000
C	-1.288876000	3.034621000	3.636267000
C	3.092502000	1.866217000	5.390948000
C	-1.378951000	1.789174000	3.034496000
C	3.001406000	0.620419000	4.790179000
C	-0.377907000	0.706586000	-0.174770000
C	4.000004000	-0.466697000	1.578637000
C	1.033537000	3.465247000	-1.871457000
C	5.412598000	2.296200000	-0.109194000
C	0.910388000	6.254238000	0.294923000
C	5.293986000	5.091532000	2.051724000
C	-0.582141000	5.220423000	3.326456000
C	3.799532000	4.052337000	5.081165000
C	-0.131996000	0.034434000	2.160480000
C	2.032572000	-0.546177000	3.029555000
C	1.571282000	1.134181000	-1.357603000
C	3.736884000	0.556294000	-0.488294000
C	2.524987000	5.078853000	-1.111721000
C	4.688057000	4.500719000	-0.238322000
C	1.409277000	6.416216000	2.556586000
C	3.576650000	5.839677000	3.425696000
C	-0.232757000	3.296979000	4.580774000
C	1.933460000	2.719564000	5.447310000
C	-0.417515000	0.765851000	3.357986000
C	1.748216000	0.186989000	4.226719000
C	0.959374000	0.237636000	-0.425960000

C	3.123983000	-0.340370000	0.442001000
C	2.429801000	3.791928000	-1.731757000
C	4.587646000	3.212515000	-0.849795000
C	1.964523000	6.517315000	1.240299000
C	4.131297000	5.942112000	2.109685000
C	0.203493000	4.647423000	4.389413000
C	2.369810000	4.069745000	5.255921000
C	12.534776000	1.971350000	-4.564153000
H	13.112345000	2.372225000	-3.726338000
H	12.764354000	0.917946000	-4.715361000
H	12.779857000	2.554325000	-5.455571000
H	-3.959822000	-1.746352000	1.251548000
H	-4.111543000	-0.593034000	-0.898269000
H	-2.776054000	9.044619000	1.532953000
H	-3.171095000	8.072145000	-0.674621000
H	-0.569622000	9.855718000	2.258651000
H	-2.020730000	-3.114726000	1.898719000
H	1.062773000	-4.047515000	1.934451000
H	3.409621000	-4.031594000	1.349674000
H	6.103910000	-3.078297000	-0.126086000
H	7.439485000	-1.533633000	-1.472524000
H	8.568185000	-1.010106000	-3.701797000
H	9.304614000	5.627952000	-3.516768000
H	8.302257000	6.218592000	-1.257179000
H	7.346550000	7.921600000	0.216496000
H	4.928453000	9.401343000	1.774202000
H	2.649940000	9.969321000	2.356791000
H	5.113973000	1.498203000	-3.157361000
H	5.373663000	3.919717000	-3.151684000
H	4.820307000	5.536307000	-4.765134000
H	2.551048000	6.340579000	-4.856907000
H	0.106617000	6.939695000	-4.211682000
H	-1.969659000	7.260711000	-3.014392000
H	-2.745786000	0.033179000	-3.202991000
H	-0.651352000	-0.092149000	-4.406335000
H	1.862344000	-0.069763000	-5.041975000
H	4.258625000	0.161824000	-4.915951000

Optimized structure of **7** with C_{70}, second conformation

(most favorable)

E=-5366.15625858227

C	-6.690384000	-3.619411000	-4.350202000
C	-7.118451000	-4.369064000	-3.267409000
C	-6.309334000	-5.344106000	-2.619681000
C	-5.059454000	-5.619668000	-3.131620000
C	-4.680336000	-4.886442000	-4.301487000
C	-5.392731000	-3.841547000	-4.883080000
C	-3.326435000	-5.096604000	-4.601957000

C	-2.541135000	-4.219014000	-5.349831000
C	-3.294166000	-3.197768000	-6.020221000
C	-4.654495000	-3.039940000	-5.818841000
C	-2.855046000	-6.012300000	-3.637831000
C	-1.520552000	-6.086682000	-3.420312000
C	-0.675448000	-5.232942000	-4.161062000
C	-1.087840000	-4.296345000	-5.108513000
C	0.523289000	-5.152849000	-3.433058000
C	1.481773000	-4.185766000	-3.726064000
C	1.179071000	-3.360370000	-4.866295000
C	-0.049904000	-3.388786000	-5.502474000
C	-3.819892000	-6.322743000	-2.659217000
C	-3.297031000	-6.824387000	-1.462513000
C	-1.883502000	-6.905665000	-1.232575000
C	-0.956432000	-6.485873000	-2.193342000
C	0.434432000	-5.929063000	-2.233557000
C	1.498954000	-5.820967000	-1.363867000
C	2.549499000	-4.923624000	-1.674659000
C	2.554396000	-4.075066000	-2.781798000
C	3.521970000	3.169088000	-2.314767000
C	3.699572000	3.837065000	-1.103166000
C	2.957238000	4.975531000	-0.704904000
C	2.049822000	5.520651000	-1.587827000
C	0.913990000	6.496529000	-1.524323000
C	0.103201000	7.096217000	-0.553792000
C	-1.232457000	7.526732000	-0.850525000
C	-1.793210000	7.369886000	-2.123467000
C	1.017190000	3.658229000	-5.170009000
C	2.123052000	3.158831000	-4.502876000
C	2.596243000	3.713498000	-3.262009000
C	1.973827000	4.897230000	-2.874380000
C	0.303321000	4.810141000	-4.701101000
C	0.923040000	5.446122000	-3.625937000
C	0.347891000	6.445417000	-2.812712000
C	-0.911573000	6.854318000	-3.092823000
C	-3.412200000	4.913385000	-5.700951000
C	-2.064592000	4.619851000	-5.825132000
C	-1.071201000	5.251308000	-5.005546000
C	-1.580108000	6.255443000	-4.181328000
C	-3.915527000	5.820564000	-4.707547000
C	-2.945898000	6.490027000	-3.967439000
C	-3.153645000	7.184977000	-2.732543000
C	-4.462591000	7.304325000	-2.315801000
C	-5.494659000	6.742031000	-3.118204000
C	-5.253155000	6.006951000	-4.266491000
C	5.521516000	1.635799000	-2.185556000
C	4.155336000	1.845807000	-2.487775000
C	3.370465000	0.757393000	-2.826446000

C	3.886767000	-0.553977000	-2.806463000
C	5.248808000	-0.755671000	-2.461492000
C	6.060788000	0.363885000	-2.184971000
C	3.059832000	-1.676214000	-2.999710000
C	3.528623000	-2.964974000	-2.813391000
C	4.896746000	-3.153727000	-2.505980000
C	5.743656000	-2.072775000	-2.349524000
C	7.167038000	-2.302208000	-2.017199000
N	7.948028000	-1.153932000	-1.808459000
C	7.485857000	0.168487000	-1.850183000
O	8.248519000	1.092962000	-1.615493000
O	7.646297000	-3.421362000	-1.925801000
C	-5.415991000	0.126823000	2.388073000
C	-5.716127000	-0.311475000	-1.751461000
C	-1.916385000	0.246733000	-3.382246000
C	0.730571000	1.029563000	-0.248021000
C	-1.432528000	0.955214000	3.314676000
C	-2.848126000	0.717480000	3.597715000
C	-6.097517000	-0.117486000	1.116295000
C	-4.721008000	-0.224307000	-2.820992000
C	-0.623791000	0.545356000	-2.766299000
C	0.540365000	1.127254000	1.198589000
C	-3.993729000	4.281350000	0.818338000
C	-2.554207000	-3.453955000	1.532433000
C	-4.666456000	4.038125000	-0.438247000
C	-3.226815000	-3.695504000	0.276235000
C	-3.682992000	4.121952000	-1.492862000
C	-2.243762000	-3.609042000	-0.779865000
C	-2.403967000	4.416123000	-0.890276000
C	-0.963182000	-3.317749000	-0.176178000
C	-2.595032000	4.516097000	0.537890000
C	-1.154737000	-3.220199000	1.253005000
C	-4.361410000	3.558519000	1.950189000
C	-3.192589000	-2.721664000	2.530501000
C	-5.679073000	3.085741000	-0.509818000
C	-4.509296000	-3.194006000	0.070468000
C	-3.754048000	3.251969000	-2.574910000
C	-2.587307000	-3.025204000	-1.994000000
C	-1.249414000	3.828595000	-1.395296000
C	-0.079837000	-2.451912000	-0.814546000
C	-1.622216000	4.019542000	1.401767000
C	-0.453642000	-2.262475000	1.983022000
C	-5.403642000	2.564758000	1.868337000
C	-4.516932000	-2.197109000	2.309224000
C	-5.743088000	2.179110000	-1.629526000
C	-4.856539000	-2.582880000	-1.189039000
C	-2.556545000	2.636873000	-3.088875000
C	-1.673384000	-2.122980000	-2.646106000

C	-0.250038000	3.306896000	-0.496255000
C	0.637043000	-1.456606000	-0.055566000
C	-2.008971000	3.263002000	2.567840000
C	-1.123499000	-1.499325000	3.007199000
C	-3.350121000	3.037216000	2.836039000
C	-2.463876000	-1.724438000	3.275842000
C	-6.048464000	2.332918000	0.663871000
C	-5.161741000	-2.428010000	1.103966000
C	-4.800216000	2.261721000	-2.642154000
C	-3.914146000	-2.499610000	-2.199449000
C	-1.330161000	2.919506000	-2.510418000
C	-0.448652000	-1.841964000	-2.065934000
C	-0.430750000	3.401722000	0.874195000
C	0.455406000	-1.364046000	1.315593000
C	-5.045340000	1.453854000	2.712711000
C	-4.607275000	-0.900224000	2.931040000
C	-6.168739000	0.887883000	-1.150889000
C	-5.729928000	-1.466873000	-0.933106000
C	-2.868781000	1.288239000	-3.487892000
C	-2.431297000	-1.066388000	-3.264807000
C	0.293016000	2.100296000	-1.064033000
C	0.724893000	-0.253852000	-0.842331000
C	-1.052366000	2.204040000	2.766185000
C	-0.614851000	-0.151443000	2.983070000
C	-3.777330000	1.745681000	3.310183000
C	-3.339241000	-0.608508000	3.528326000
C	-6.358088000	0.983471000	0.265604000
C	-5.918513000	-1.370760000	0.483073000
C	-4.254672000	1.055273000	-3.210618000
C	-3.815219000	-1.298423000	-2.986555000
C	-0.375928000	1.860862000	-2.307332000
C	0.051828000	-0.491856000	-2.082611000
C	-0.078164000	2.289015000	1.720229000
C	0.359446000	-0.067118000	1.937444000
C	9.369302000	-1.324249000	-1.480979000
H	9.569810000	-0.931071000	-0.480223000
H	9.600289000	-2.387230000	-1.525592000
H	9.978792000	-0.763993000	-2.194477000
H	-8.102931000	-4.169075000	-2.853922000
H	-7.329456000	-2.838177000	-4.754224000
H	-6.520359000	6.853588000	-2.778263000
H	-6.080922000	5.539582000	-4.793912000
H	-4.724671000	7.778303000	-1.373242000
H	-6.675144000	-5.816756000	-1.711793000
H	-3.947361000	-7.050949000	-0.621300000
H	-1.561705000	-7.189042000	-0.233357000
H	1.524711000	-6.349797000	-0.414717000
H	3.345815000	-4.806796000	-0.944123000

H	5.302642000	-4.153874000	-2.383606000
H	6.167045000	2.475504000	-1.944120000
H	4.377591000	3.395193000	-0.377575000
H	3.077750000	5.354414000	0.306494000
H	0.417860000	7.144158000	0.485718000
H	-1.835780000	7.869581000	-0.013503000
H	2.009420000	-1.511944000	-3.209945000
H	2.312574000	0.896025000	-3.029149000
H	2.614927000	2.280524000	-4.910184000
H	0.669349000	3.129438000	-6.053791000
H	-1.770022000	3.846281000	-6.529975000
H	-4.121500000	4.371355000	-6.321951000
H	-5.163249000	-2.221011000	-6.321743000
H	-2.783090000	-2.485680000	-6.663389000
H	-0.232938000	-2.671066000	-6.298139000
H	1.919558000	-2.646422000	-5.213829000

Optimized structure of complex **7** with C_{70}, third conformation.

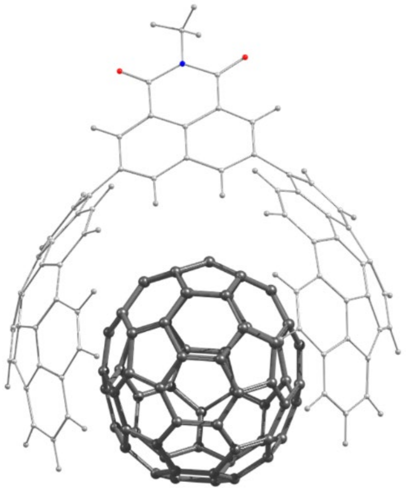

E=-5366.15247322445

C	-3.039157000	-1.167524000	-0.863991000
C	-2.985608000	-1.836454000	0.346930000
C	-1.914448000	-2.695288000	0.720941000
C	-0.907984000	-2.940563000	-0.188129000
C	-1.038683000	-2.302278000	-1.464039000
C	-2.002477000	-1.362054000	-1.816298000
C	0.132234000	-2.458047000	-2.219820000
C	0.515262000	-1.616479000	-3.264422000
C	-0.514886000	-0.708140000	-3.680116000
C	-1.721424000	-0.609857000	-3.007277000
C	0.997755000	-3.243859000	-1.429344000
C	2.322046000	-3.215509000	-1.706130000
C	2.765502000	-2.395366000	-2.765129000
C	1.959841000	-1.583294000	-3.563167000
C	4.135180000	-2.184485000	-2.539568000
C	4.840247000	-1.210226000	-3.242176000

C	4.073611000	-0.509366000	-4.238018000
C	2.703672000	-0.661360000	-4.370686000
C	0.478334000	-3.516058000	-0.149086000
C	1.438566000	-3.864021000	0.807706000
C	2.842054000	-3.837102000	0.513876000
C	3.322161000	-3.460158000	-0.745830000
C	4.552072000	-2.839836000	-1.336777000
C	5.849344000	-2.599761000	-0.937393000
C	6.642618000	-1.701742000	-1.692565000
C	6.176956000	-0.971175000	-2.786030000
C	6.919258000	6.243417000	-2.909037000
C	7.527386000	6.873833000	-1.823049000
C	6.913248000	7.883287000	-1.041783000
C	5.663521000	8.338052000	-1.403895000
C	4.561990000	9.136114000	-0.771972000
C	4.169598000	9.558076000	0.503394000
C	2.794275000	9.816786000	0.823655000
C	1.775081000	9.664811000	-0.123240000
C	3.419308000	6.560672000	-4.434458000
C	4.746065000	6.176204000	-4.330952000
C	5.636547000	6.716983000	-3.337676000
C	5.118892000	7.782916000	-2.606459000
C	2.854501000	7.576787000	-3.594695000
C	3.799783000	8.223249000	-2.798712000
C	3.519675000	9.083016000	-1.716517000
C	2.224787000	9.333024000	-1.415839000
C	-0.950238000	7.304071000	-3.033760000
C	0.253538000	7.180311000	-3.708615000
C	1.440954000	7.859296000	-3.275669000
C	1.226442000	8.734178000	-2.210088000
C	-1.080634000	8.065910000	-1.823194000
C	0.050999000	8.784052000	-1.448672000
C	0.304954000	9.357423000	-0.160171000
C	-0.734747000	9.301198000	0.743475000
C	-1.957067000	8.688487000	0.348140000
C	-2.140353000	8.069309000	-0.876788000
C	8.890290000	4.818470000	-3.631761000
C	7.505007000	4.974849000	-3.390620000
C	6.693677000	3.854888000	-3.421317000
C	7.228234000	2.560191000	-3.553517000
C	8.626004000	2.410712000	-3.746493000
C	9.435180000	3.563760000	-3.830940000
C	6.438106000	1.414303000	-3.339547000
C	7.001846000	0.152084000	-3.276916000
C	8.386620000	0.009568000	-3.531175000
C	9.180905000	1.113916000	-3.777037000
C	10.629740000	0.935830000	-4.016881000
N	11.381205000	2.112392000	-4.170798000

C	10.885341000	3.419756000	-4.069956000
O	11.641251000	4.372696000	-4.179198000
O	11.153412000	-0.165242000	-4.079102000
C	0.070167000	0.015458000	1.131040000
C	4.100856000	0.184169000	2.191618000
C	5.042368000	4.170690000	3.010312000
C	1.595415000	6.454367000	2.452956000
C	-1.482924000	3.883943000	1.290700000
C	-1.523559000	2.437457000	1.070903000
C	1.403859000	-0.520588000	1.396205000
C	4.964704000	1.302670000	2.575970000
C	4.242163000	5.396043000	2.981106000
C	0.235688000	6.092819000	2.051517000
C	1.851968000	2.838023000	-2.119045000
C	-0.094391000	1.742094000	5.457128000
C	3.169365000	2.309437000	-1.849003000
C	1.225425000	1.213707000	5.719804000
C	4.018788000	3.411070000	-1.464065000
C	2.080491000	2.316194000	6.099147000
C	3.233895000	4.621759000	-1.501296000
C	1.289334000	3.526036000	6.070001000
C	1.892333000	4.267700000	-1.902901000
C	-0.054618000	3.171399000	5.673276000
C	0.729344000	2.109986000	-1.738340000
C	-0.851641000	1.222398000	4.410963000
C	3.311625000	1.076860000	-1.218849000
C	1.730991000	0.187788000	4.925485000
C	4.981934000	3.233824000	-0.475341000
C	3.403822000	2.344948000	5.667985000
C	3.437570000	5.604236000	-0.537806000
C	1.855595000	4.712677000	5.611931000
C	0.807243000	4.906825000	-1.313714000
C	-0.775024000	4.018109000	4.835271000
C	0.881824000	0.841131000	-1.071215000
C	-0.320026000	0.165036000	3.586271000
C	4.302347000	0.898024000	-0.187562000
C	3.100640000	0.226511000	4.475470000
C	5.183427000	4.250728000	0.524357000
C	3.982724000	3.574951000	5.186145000
C	2.308422000	6.258449000	0.073480000
C	1.108461000	5.581896000	4.736699000
C	-0.352918000	4.148144000	-0.915667000
C	-1.552190000	3.474472000	3.748365000
C	-0.391829000	2.777943000	-1.123841000
C	-1.589075000	2.104844000	3.540376000
C	2.144968000	0.331806000	-0.820826000
C	0.945258000	-0.342104000	3.838448000
C	5.120105000	1.956747000	0.177159000

C	3.919845000	1.283562000	4.839157000
C	4.425103000	5.411048000	0.494945000
C	3.224648000	4.734527000	5.158628000
C	1.020926000	5.914478000	-0.304778000
C	-0.180247000	5.241750000	4.356275000
C	-0.148563000	0.720274000	-0.074840000
C	-0.742937000	0.386505000	2.227726000
C	3.753610000	0.034632000	0.827227000
C	3.159095000	-0.296550000	3.134042000
C	5.455893000	3.602742000	1.781949000
C	4.861627000	3.268976000	4.086543000
C	2.607230000	6.483074000	1.464022000
C	2.014199000	6.148917000	3.770020000
C	-0.857597000	4.698734000	0.316405000
C	-1.452936000	4.366510000	2.621442000
C	-0.936657000	1.915110000	-0.106387000
C	-1.529445000	1.581898000	2.199243000
C	2.420827000	-0.313162000	0.435437000
C	1.827208000	-0.645919000	2.740256000
C	5.418233000	2.186579000	1.567701000
C	4.823045000	1.853618000	3.872300000
C	3.914942000	5.961878000	1.724843000
C	3.321592000	5.626675000	4.031546000
C	-0.007681000	5.786113000	0.693023000
C	-0.604685000	5.457696000	2.996738000
C	12.823237000	1.993515000	-4.421038000
H	13.381571000	2.454358000	-3.601366000
H	13.064819000	0.934885000	-4.499542000
H	13.079676000	2.519368000	-5.344367000
H	-3.771791000	-1.657159000	1.074879000
H	-3.846731000	-0.466430000	-1.059434000
H	-2.767945000	8.660823000	1.070580000
H	-3.073741000	7.553023000	-1.086941000
H	-0.634914000	9.674090000	1.759644000
H	-1.888945000	-3.099168000	1.729902000
H	1.155539000	-4.048179000	1.841218000
H	3.523153000	-4.004768000	1.344657000
H	6.262568000	-3.028783000	-0.028418000
H	7.644647000	-1.485467000	-1.331195000
H	8.850035000	-0.972932000	-3.524704000
H	9.549700000	5.681656000	-3.653598000
H	8.482693000	6.482999000	-1.482426000
H	7.412592000	8.224521000	-0.138892000
H	4.880965000	9.587906000	1.324968000
H	2.562179000	10.022149000	1.865778000
H	5.383417000	1.545250000	-3.119362000
H	5.633675000	3.958167000	-3.222132000
H	5.107255000	5.397838000	-4.996766000

H	2.790282000	6.043296000	-5.154560000
H	0.299984000	6.501655000	-4.556617000
H	-1.805601000	6.734263000	-3.388973000
H	-2.446303000	0.127273000	-3.344266000
H	-0.332441000	-0.030887000	-4.510702000
H	2.185352000	-0.028909000	-5.087095000
H	4.573198000	0.212019000	-4.877873000

8. Reference

1. J. Y. Xue, W. Nakanishi, D. Tanimoto, D. Hara, Y. Nakamura, H. Isobe, Tetrahedron Letters, 2013, 54, 4963–4965.
2. O. Papaianina, V. A. Akhmetov, A. A. Goryunkov, F. Hampel, F. W. Heinemann and K. Y. Amsharov, Angew. Chem. Int. Ed. Engl., 2017, 56, 4834–4838.
3. B. Scholz, A. S. Oshchepkov, O. Papaianina, C. Ruppenstein, V. A. Akhmetov, D. I. Sharapa, Prof. K. Y. Amsharov, M. E. Pérez-Ojeda, Chem. Eur. J. 2023, 29, e202302778.
4. V. Akhmetov, M. Feofanov, C. Ruppenstein, J. Lange, D. Sharapa, M. Krstić, F. Hampel, E. A. Kataev, and K. Y. Amsharov, Chem. Eur. J. 2022, 28, e202200584.
5. K.W. Kim, S. H. Kwon, B. J. Lee, S. O. Ahn, J. H. Lee, J. H. Choi, Dyes and Pigments, 2020, 181, 108617.
